# The Effect of Organic Materials on the Response of the Soil Microbiome to Bisphenol A

**DOI:** 10.3390/molecules30193868

**Published:** 2025-09-24

**Authors:** Magdalena Zaborowska, Jadwiga Wyszkowska, Mirosława Słaba, Agata Borowik, Jan Kucharski, Przemysław Bernat

**Affiliations:** 1 Department of Soil Science and Microbiology, University of Warmia and Mazury in Olsztyn, Plac Łódzki 3, 10-727 Olsztyn, Poland; agata.borowik@uwm.edu.pl (A.B.); jan.kucharski@uwm.edu.pl (J.K.); 2 Department of Industrial Microbiology and Biotechnology, Faculty of Biology and Environmental Protection, University of Lodz, 90-237 Lodz, Poland; miroslawa.slaba@biol.uni.lodz.pl (M.S.); przemyslaw.bernat@biol.uni.lodz.pl (P.B.)

**Keywords:** bisphenol A (BPA), soil microbial diversity, organic sorbents, soil phospholipids, ergosterol

## Abstract

In view of the increasing environmental pollution caused by bisphenol A (BPA), understanding its impact on the microbiological properties of soil, which play a key role in maintaining soil fertility and consequently ecosystem stability, is particularly important. Therefore, the aim of this study was to assess the sensitivity of the soil microbiome to this xenobiotic and to evaluate the potential of organic materials such as starch (St), grass compost (Co), and fermented bark (B) to restore the balance of soil cultivated with *Zea mays*. The negative effects of BPA on the abundance, diversity, and structure of bacterial and fungal communities in soil contaminated with 500 and 1000 mg kg^−1^ d.m. of soil were confirmed. Changes in the phospholipid profile, including phosphatidylethanolamine (PE), phosphatidylcholine (PC), phosphatidylglycerol (PG), and ergosterol (E), were also assessed. BPA applied at 1000 mg kg^−1^ d.m. of soil inhibited the proliferation of organotrophic bacteria and actinomycetes, while stimulating fungal growth. This xenobiotic’s impact is also reflected by a decrease in PC and PG levels in soil under BPA pressure. Through amplification of the V3-V4 16S rRNA region (for bacteria) and the ITS1 region (for fungi), the dominant bacterial phylum Proteobacteria was identified, with genera including *Cellulosimicrobium*, *Caulobacter*, *Rhodanobacter*, *Sphingomonas*, *Mucilaginibacter*, and *Pseudomonas*. Among fungi, Ascomycota dominated, primarily represented by the genus *Penicillium*. Of all the organic materials tested for mitigating BPA’s negative effects, grass compost was identified as the most promising, not only restoring soil homeostasis but also enhancing the growth and development of *Zea mays* cultivated in BPA-contaminated soil.

## 1. Introduction

The Anthropocene epoch accurately defines the period in which humanity currently exists. It refers to the spectacular consequences of human activity, mainly the disruption of ecosystem biodiversity and biogeochemical cycles, and increasing pollution of the environment with xenobiotics [1]. In search of solutions, new trends and innovative programs are emerging, whose primary goal is to maintain or increase agricultural productivity and profitability stabilized by the application of sustainable development principles [2]. The Climate Smart Agriculture (CSA) program addresses such challenges by focusing on solving global food security issues and paying particular attention to the importance of the soil microbiome in ensuring carbon sequestration and thus climate regulation [3]. Another initiative is the “4 per 1000 soils for food security and climate,” stimulated by the vision of soils rich in soil organic carbon (SOC), whose reserves are increasing by 4‰ per year, to ultimately end hunger by 2050 [4,5]. Also important is the continuing trend of regenerative agriculture, defined as a “back to basics” solution to improve soil condition as measured by biodiversity [6]. This stems from an awareness of environmental degradation across much of the world. The initiative currently covers 72.3 million hectares of agricultural land in 187 countries and is referred to as the phenomenon of ‘ecological change’ [7].

Plastics, including bisphenol A (BPA), are currently considered to be the main cause of environmental degradation [8,9]. This is referenced in the 2022 resolution on an international agreement aimed at eliminating the plastic crisis, which was supported by the United Nations Environment Assembly (UNEA) [10]. One of the important tools established under management decisions are multimedia environmental models (MEN), which are designed to describe the behavior of chemicals in the environment, including BPA [11]. It is estimated that the BPA market will reach 8.72 million tonnes by 2025, growing to 11.98 million tonnes by 2030. Annual production growth over this period is forecast to remain at 6.56% [12]. However, consumption patterns in different economic sectors are changing. Currently, the dominant sources of BPA are the automotive sector, construction, and the expansion of digital infrastructure, whereas previously it was concerned with optics and plastic packaging [10]. This significant transformation is mainly driven by technological advances and the growing demand for epoxy resins, which are essential for producing electrical and electronic components and are also widely used in construction for producing adhesives, paints, and sealants. Notably, in 2024, demand for polycarbonate resins accounted for 62% of global BPA consumption [12]. More worryingly, Chen et al. [13] proved that microplastics, one of the most polluting substances in recent years, transport BPA around the environment.

Nevertheless, restrictions on the use of this phenolic compound are primarily due to its detrimental impact on human health. Significant consequences of its toxicity include neurobehavioral disorders, as well as disorders of the reproductive and immune systems. However, the most concerning effect of this compound spreading in the environment is an increased risk of autism spectrum disorder (ASD) [14,15], depression and cancer [16,17].

Numerous reports on the spread of BPA in the air [18,19] and water [9,20,21] exacerbate social unrest. The compound enters the soil through improper electronic waste disposal practices [22,23] and the use of biosolids [24] or pesticides [25].

The toxicity of BPA results from the instability and bioavailability of the non-extractable soil residues of this compound [26]. The adsorption of bisphenols onto soil matrices and their subsequent transformation and migration are responsible for their physicochemical properties, including the presence of hydroxyl groups (-OH), as well as their interaction with dissolved organic matter (DOM). This interaction occurs through hydrogen bonds involving binding with carboxyl groups (-COOH) and hydroxyl (-OH) groups, as well as hydrophobic bonds [27]. These interactions are also supported by the Freundlich affinity constant (Kf). Ou et al. [28] demonstrated that higher values of this parameter in soils rich in organic matter indicate better BPA sorption capacity.

Therefore, the use of adsorbents for BPA removal is well justified. A current trend is the application of organic sorbents, which are attractive due to their cost-effectiveness, chemical stability, and the presence of a wide range of functional groups that enhance their efficiency. Moreover, they are abundant in nature [29]. Another important factor in their selection is that this strategy aligns with all initiatives aimed at preserving soil condition and biodiversity, owing to the adsorbents’ renewability and biodegradability [30].

Compost is a lignocellulosic resource that is rich in carbohydrates, proteins, fats, and lignin. It generates its effectiveness in the decomposition and immobilization of organic pollutants owing to the release of catabolic enzymes [31]. The effectiveness of using compost as a biostimulating substance is also confirmed by reports from researchers indicating a significant reduction in the organic carbon (C_org_) content of topsoil in arable land [32]. According to Supriyadi et al. [33], the wood processing sector produced around 200 million m^3^ of bark waste in 2021, which presents many logistical and environmental disposal challenges. Therefore, it is worth considering bark as an adsorbent of organic pollutants, given its porosity resulting from a particle density of 1.2 g cm^−3^ [34]. The adsorption of BPA in tree bark results from van der Waals forces associated with the complex structure of its components, including lignin, which contains numerous phenylpropanoid units, such as p-hydroxyphenyl, syringyl, and guaiacyl, that promote such interactions [33,35]. Interest in starch as an adsorbent stems from the fact that it is a glucose polymer commonly found in the environment. These are linked by α-D-(1,4) and α-D-1,6 bonds [36]. It is also worth noting that the starch production and use sector was valued at USD 13.1 million in 2023 [37]. Most importantly, Kohli et al. [38] demonstrated that cross-linked starch polymers have the potential to absorb organic pollutants.

Biological indicators, including microbiological activity are among the most sensitive parameters for determining soil quality [39]. Therefore, it is essential to verify this parameter in soils used for agricultural purposes, particularly those exposed to BPA. Addressing this issue involves the biodegradation potential of microorganisms with respect to bisphenols, attributable to the activity of extradiol dioxygenases and monooxygenases [40]. In turn, fungi, in addition to laccase and cytochrome P-450, are equipped with triphenylmethane reductase, polyketide synthase, and two peroxidases: lignin and manganese [41,42,43]. Soil lipids can also be used to indicate soil health and microbial diversity. Phospholipids found in soil primarily originate from the cell membranes of living microorganisms. They decompose rapidly after cell death, which makes them a reliable indicator of active soil microbiota. Approximately 20% of soil phospholipids derive from other sources, such as plant roots, dead plant fragments, and dead cells. These phospholipids can be released into the soil and subsequently adsorbed by soil organic matter [44].

The research objectives were established based on observed trends and the implementation of solutions consistent with sustainable development principles. These include: the response of the soil microbiome to bisphenol A (BPA), with particular emphasis on changes in community structure, and phospholipid and ergosterol content in the soil (1), and the selection of the organic compound with the highest bioremediation potential from among three organic substances: grass compost, fermented bark, and starch, which was an innovative aspect of our research (2). Two research hypotheses were put forward. The first relates to the toxicity of BPA, which will significantly disrupt the soil balance, and the scale of this phenomenon will be reflected in the reaction of the microbiome and the response of the cultivated plant (*Zea mays*). The second hypothesis assumes that the organic substances used will improve the condition of the soil to varying degrees, which will give the research a practical dimension, demonstrating its potential for application in agriculture and environmental protection.

## 2. Results

### 2.1. Plant

The experiment analyzed the response of *Zea mays* to BPA pressure (Figure S1). It was observed that the plant exhibited a progressive disturbance in its growth and development in response to the increasing level of soil contamination with this xenobiotic. Exposure to 500 mg BPA kg^−1^ d.m. of soil (C_500) resulted in a 44.11% reduction in the yield of the aboveground part of *Zea mays*, while exposure to 1000 mg BPA kg^−1^ d.m. of soil (C_1000) caused a reduction of as much as 69.75% compared to the control. It is also important to emphasize that the root biomass of plants decreased to a greater extent in the BPA-contaminated treatments. In the parallel samples (C_500) and (C_1000), the values of this parameter decreased by 57.74% and 86.11%, respectively, and corresponded to the increasing P/R values (C_500: 4.64; C_1000: 7.59) in these treatments. These relationships were further emphasized by the IF_BPA values determined in the study, with the lowest values (IF_BPA_ = −0.861) for the roots of *Zea mays* and (IF_BPA_ = −0.699) observed for the aboveground part of the plant in the treatments with the highest BPA dose. Of the three organic materials that were examined, compost was found to be the most effective (Figure S1). In addition, it was discovered that compost significantly mitigated the inhibitory effect of BPA on the growth and development of *Zea mays*, irrespective of the level of contamination with this phenolic compound. It is noteworthy that there was a strong stimulation of root growth and development in soil amended with grass compost in combination with 1000 mg BPA kg^−1^ d.m. of soil, manifested by an increase in root biomass by as much as 83.42% compared to the control. This tendency was further substantiated by the IF_S_ value of 0.835 for root biomass. The efficacy of starch in mitigating the inhibitory effect of 1000 mg BPA kg^−1^ d.m. of soil (St_1000) was demonstrated. In this particular treatment, the IF_S_ values were determined to be 0.259 for the aboveground part of the plant and 0.802 for root biomass. However, the application of fermented bark did not yield the anticipated outcomes.

### 2.2. Culturable Bacteria

The presence of bisphenol A (BPA) in soil has been shown to induce substantial alterations in the prevalence of all the analyzed groups of microorganisms, with the interaction of individual groups with this phenolic compound exhibiting variation (Table S1, Figure S2). The most sensitive organisms to the xenobiotic applied to the soil were organotrophic bacteria (Org), while actinomycetes (Act) were less affected. The present study demonstrated that fungi (Fun) exhibited a positive response to BPA. With regard to organotrophic bacteria, increasing levels of BPA contamination in the soil resulted in inhibition rates of 4.81% (500 mg BPA kg^−1^ d.m. of soil) and 34.32% (1000 mg BPA kg^−1^ d.m. of soil) as compared to the control. The toxicity of the xenobiotic used against actinomycetes was manifested by a 16.68% reduction in the abundance of this group after exposure to a lower dose of BPA and a 9.30% reduction after exposure to 1000 mg of the phenolic compound per kg of soil. Conversely, the IF_BPA_ indices exhibited a contrasting response in fungi upon exposure (Figure S2). This was demonstrated by a threefold increase in the abundance of this group of microorganisms under 500 mg of BPA kg^−1^ d.m. of soil pressure, corresponding to an IF_BPA_ value of 1.738, as well as stimulation of fungal growth, resulting in an IF_BPA_ value of 0.839.

The pressure of increasing BPA levels in the soil also moderated the rate of multiplication of all groups of microorganisms. These changes were tracked using the colony development index (CD) (Table S2). Although BPA slowed down the growth of organotrophic bacteria (Org), the negative impact on this parameter resulted in a decrease in CD values in sites contaminated with this xenobiotic by 14.51% (C_500) and 12.44% (C_1000). However, the reaction of this group of microorganisms did not change from fast-growing to slow-growing in response to BPA. BPA had a greater negative effect on their growth dynamics than on organotrophic bacteria, resulting in an average 25% reduction in the CD values in both contaminated treatments. Incidentally, these treatments were assigned to a common homogeneous group. The reaction of actinomycetes was different. Although BPA did not favor their growth at either contamination level, actinomycetes represented the strategy of slow-growing microorganisms at each site, including the control site. It is worth noting that the ability of fungi to multiply in soil contaminated with BPA was not reflected in their ecophysiological diversity (EP) (Table S1). The lowest values obtained for this indicator suggest that only a few fungi are highly effective in biodegrading this phenolic compound. The opposite relationship was observed for organotrophic bacteria (Org) and actinomycetes (Act): their EP index values for these increased by 22.53% and 7.23%, respectively, compared to the control in response to 500 and 1000 mg of BPA kg^−1^ d.m. of soil.

Determining the impact factors (IFs) of organic materials on soil properties allowed us to evaluate their ability to stimulate microbial activity (Figure S2). Compost (IF_S_ = 1.727; Co_0) was the most effective at inducing organotrophic bacterial growth, whereas starch enhanced the proliferation of fungi (IF_S_ = 2.527; St_0) and actinomycetes (IF_S_ = 0.987; St_0). However, our research priorities meant that verifying the effectiveness of organic materials in neutralizing the toxic effect of BPA on the soil microbiome was more important. In soil contaminated with 500 mg of BPA kg^−1^ d.m. of soil, fermented bark (Org; IF_S_ = 1.678, Act; IF_S_ = 1.372) was the most effective organic material, while the highest IF_S_ values were obtained in soil contaminated with 1000 mg of BPA kg^−1^ d.m. of soil after enrichment with grass compost (Org; IF_S_ = 1.452, Act; IF_S_ = 0.301). It should also be emphasized that both organic materials enhanced the beneficial effect of BPA on fungi to a greater extent than starch did.

The type of organic material used also affected the rate of microbial growth and altered their ecophysiological diversity (Table S1). In the control objects, all improvers had a positive effect on the CD indices of the analyzed groups of microorganisms, except for fungi. However, only grass compost neutralized the inhibitory effect of 500 and 1000 mg BPA kg^−1^ d.m. of soil on organotrophic bacteria. Increases of 26.46% and 15.82%, respectively, were recorded compared to parallel control sites. Combining 500 mg of BPA with BPA and compost had an equally beneficial effect on the growth dynamics of fungi. The CD value (58.08) in the Co_500 object was 19.78% higher than the established index value (48.49) in the control soil (C_500). Furthermore, an increase in the ecophysiological diversity of fungi (EP) was only observed in response to compost being added to the soil, which mitigated the toxicity of BPA at both applied contamination levels.

### 2.3. Non-Culturable Bacteria

#### 2.3.1. Bacteria

**Figure 1:**
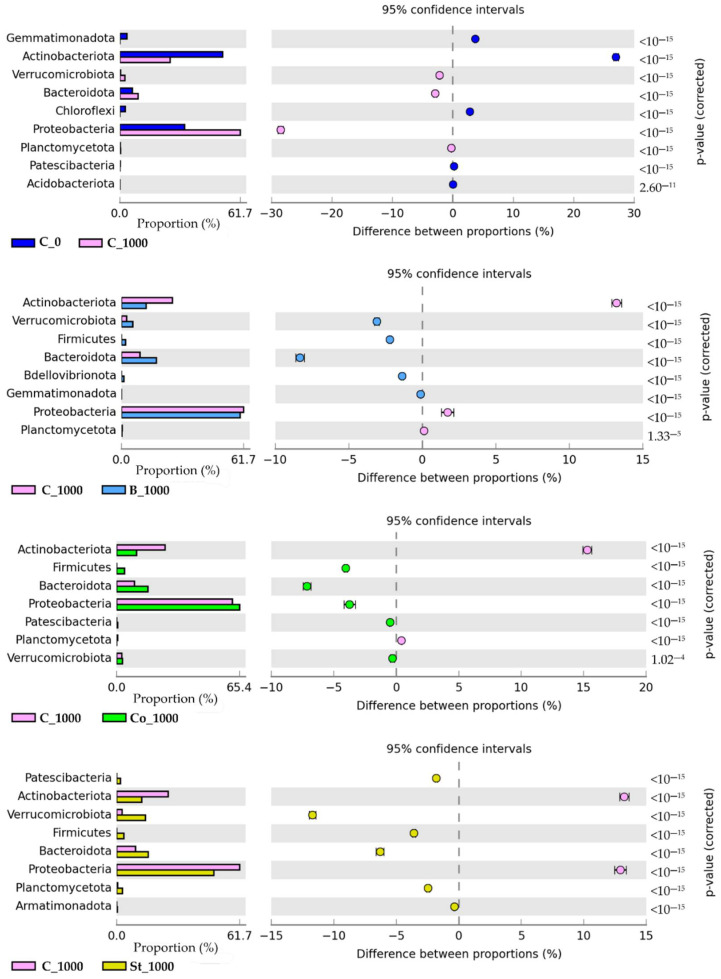
Relative abundance of dominant bacterial phyla in soils. C_0—uncontaminated soil, St—starch, Co—compost, B—fermented bark, 1000—dose of BPA kg^−1^ d.m. of soil.

Bacteria from the phyla Actinobacteriota and Proteobacteria were dominant in all soil samples (Figure 1). At the control sites, the highest ASV value was assigned to the Actinobacteriota phylum, amounting to 48,191 and accounting for 52.78% of all bacteria. By contrast, Proteobacteria dominated in BPA-contaminated soil, accounting for 61.66%. Notably, the ASV value of Proteobacteria increased by 94.72% in this group of sites, while that of Actinobacteriota decreased by 48.45% compared to the control samples. Of the three organic materials, fermented bark had a particularly beneficial effect on the regrouping of bacteria at this taxonomic level. It stimulated the greatest increase in the abundance of ASVs belonging to three phyla: Proteobacteria (67,111 ASVs), Bacteroidota (19,880 ASVs) and Verrucomicrobiota (6486 ASVs). The values of these ASVs increased by 13.77%, 54.56% and 60.08%, respectively. Furthermore, it was observed that all verified enhancers generated representatives of the Firmicutes phylum.

**Figure 2:**
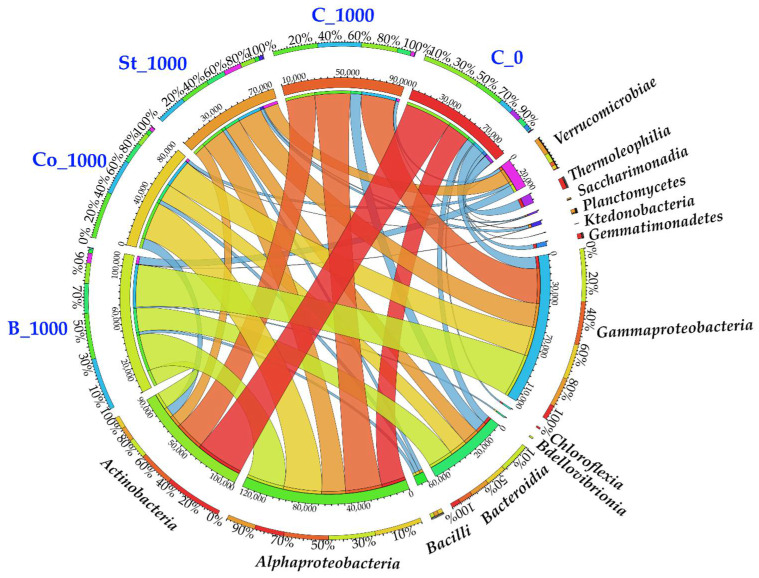
Dominant classes of bacteria in soils. C_0—uncontaminated soil, St—starch, Co—compost, B—fermented bark, 1000—dose of BPA kg^−1^ d.m. of soil.

The impact of BPA on the bacterial phylum was reflected in the ASV values established at the class level. The following taxonomic units proved to be representative, according to values of more than 1% of the sequences that were assigned: *Actinobacteria, Alphaproteobacteria*, *Gammaproteobacteria,* and *Bacteroidia* (Figure 2). Exposure to 1000 mg of BPA kg^−1^ of d.m. of soil increased the number of ASVs in the *Alphaproteobacteria* class by 50.46% and in the *Bacteroidia* class by 51.45%, compared to control samples. The largest increases in ASV values were found in *Verrucomicrobiae* and *Gammaproteobacteria* classes: from 451 to 2554 and from 10,746 to 29,578, respectively. The negative effect of this phenolic compound on certain bacterial classes, such as *Thermoleophilia*, *Chloroflexia*, *Gemmatimonadetes,* and *Saccharimonadiae*, was also observed. These were not present in BPA-contaminated sites. Additionally, the number of *Actinobacteria* ASVs decreased by 38.51% compared to the control.

**Figure 3:**
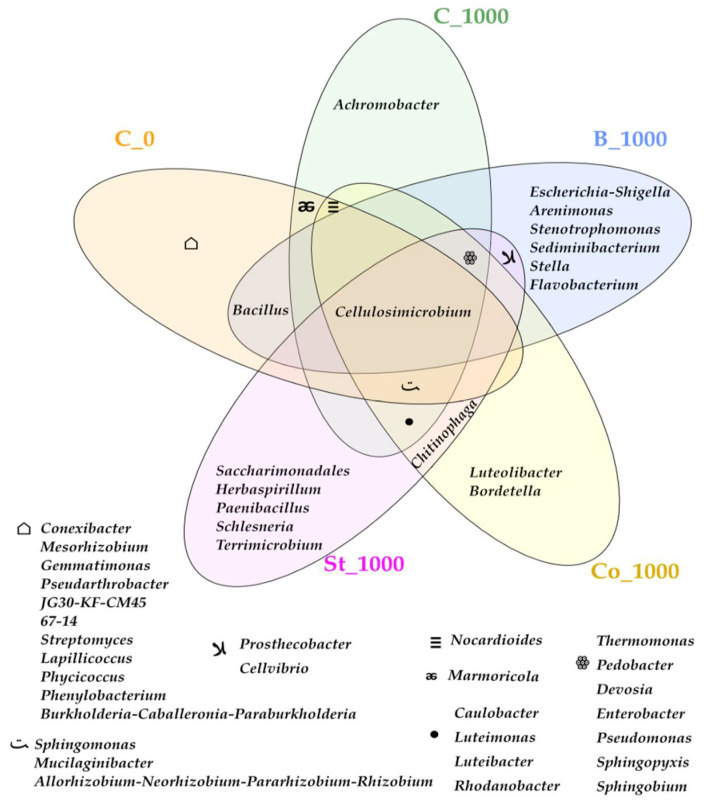
Unique and common genera of bacteria presented in the form of a Venn diagram. C_0—uncontaminated soil, St—starch, Co—compost, B—fermented bark, 1000—dose of BPA kg^−1^ d.m. of soil.

Changes in the bacterial structure at the class level were also reflected in shifts at the genus level (Figure 3). A total of 47 bacterial genera were identified in BPA-contaminated soil, but the abundance of ASVs at this taxonomic level was primarily dominated by the genus Cellulosimicrobium (19,842 ASVs), which belongs to the phylum Actinobacteriota and the class Actinobacteria. This genus accounted for 34% of all bacteria. Other genera with notable abundance included *Caulobacter* (7110 ASVs; 12.18%), *Rhodanobacter* (6793 ASVs; 11.64%), *Mucilaginibacter* (3848 ASVs; 6.59%), and *Sphingomonas* (3605 ASVs; 6.18%). However, BPA exposure reduced the relative abundance of *Sphingomonas*, *Cellulosimicrobium*, and *Mucilaginibacter* by 7.61%, 4.49%, and 3.49%, respectively, compared to the control.

Two noteworthy trends were also observed. The first indicates that BPA contributed to the elimination of eleven genera of bacteria: *Burkholderia-Caballeronia-Paraburkholderia*, *Phenylobacterium*, *Gemmatimonas*, *Pseudarthrobacter*, *JG30-KF-CM45,67-14*, *Streptomyces*, *Lapillicoccus*, *Phycicoccus*, *Mesorhizobium,* and *Conexibacter.* The second refers to soil samples contaminated with BPA, in which the following genera were found to be present: *Luteibacter*, *Rhodanobacter*, *Sphingobium*, *Thermomonas*, *Pedobacter*, *Devosia, Enterobacter*, *Pseudomonas*, *Sphingopyxis*, *Caulobacter*, *Luteimonas,* and *Achromobacter.* These genera were absent from the control sites. Metagenomic analysis identified *Achromobacter* as a unique genus belonging to the phylum Proteobacteria, class *Gammaproteobacteria*, in soil exposed to phenolic compound pressure (Figure 3). A combination of starch and soil contaminated with xenobiotics initiated the proliferation of the largest number of bacterial genera not present in the other samples. These included *Escherichia-Shigella*, *Arenimonas*, *Stenotrophomonas,* and *Stella*, which are assigned to the phylum Proteobacteria, as well as *Sediminibacterium* and *Flavobacterium*, which represent the phylum Bacteroidota.

**Figure 4:**
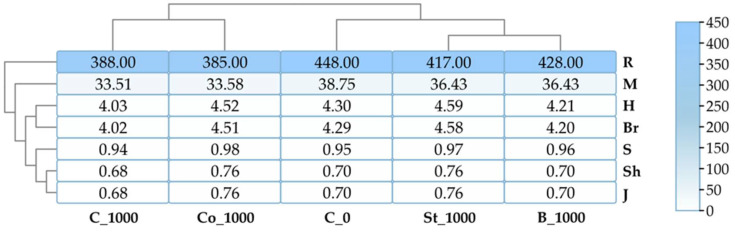
Bacterial diversity indices Shannon (H), Simpson (S), Margalef (M), Brillouin (Br), and Richness (R); Pielou (J, Shavenen Sh); C_0—uncontaminated soil, St—starch, Co—compost, B—fermented bark, 1000—dose of BPA kg^−1^ d.m. of soil.

An analysis of six specific indicators of bacterial diversity revealed that BPA negatively impacts this parameter (Figure 4). The inhibitory effect of the phenolic compound was most clearly demonstrated by the Margalef index, which showed a 13.53% reduction in diversity compared to the control. Values for all indicators increased in objects containing starch and BPA (St_1000), grass compost and BPA (Co_1000), and fermented bark and BPA (B_1000).

#### 2.3.2. Fungi

**Figure 5:**
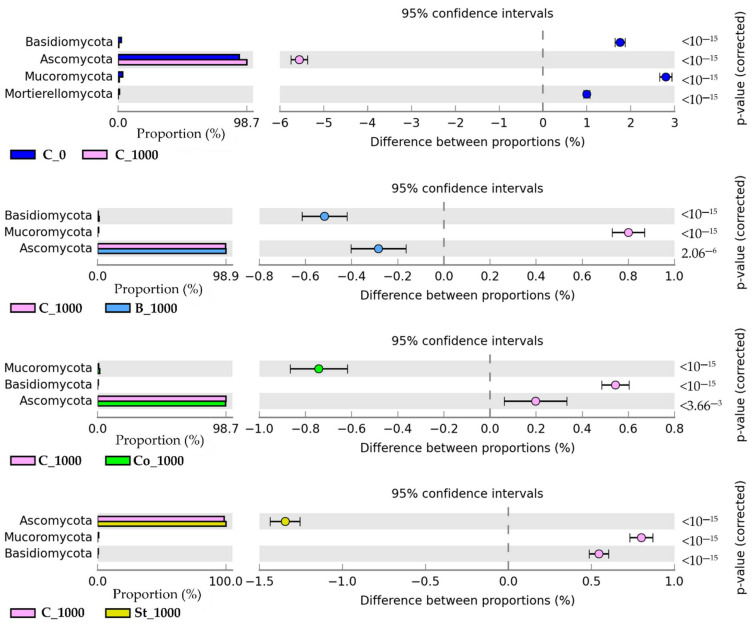
The relative abundance of dominant phyla of fungi in soils. C_0—uncontaminated soil, St—starch, Co—compost, B—fermented bark, 1000—dose of BPA kg^−1^ d.m. of soil.

Analysis of OTU data showed that the phylum Ascomycota represented mold fungi, regardless of soil use (Figure 5). The phyla Mucoromycota, Basidiomycota, and Mortierellomycota were also identified. In soil samples that were not contaminated with BPA, Ascomycota (86,613 OTUs) accounted for 93.10% of all fungi. However, exposure to 1000 mg BPA kg^−1^ d.m. of soil increased its share in this pool of objects to 98.66%, while reducing the number of OTUs by 5.97% compared to the control. Enrichment of the soil with individual organic materials moderated the abundance of the dominant phylum. The greatest increase in OTU values, of up to 98.92%, was induced by starch in combination with 1000 mg BPA kg^−1^ d.m. of soil (130,452; St_1000) compared to objects subjected to phenolic compound pressure. Compost and fermented bark did not offset the negative impact of BPA on the analyzed taxon.

**Figure 6:**
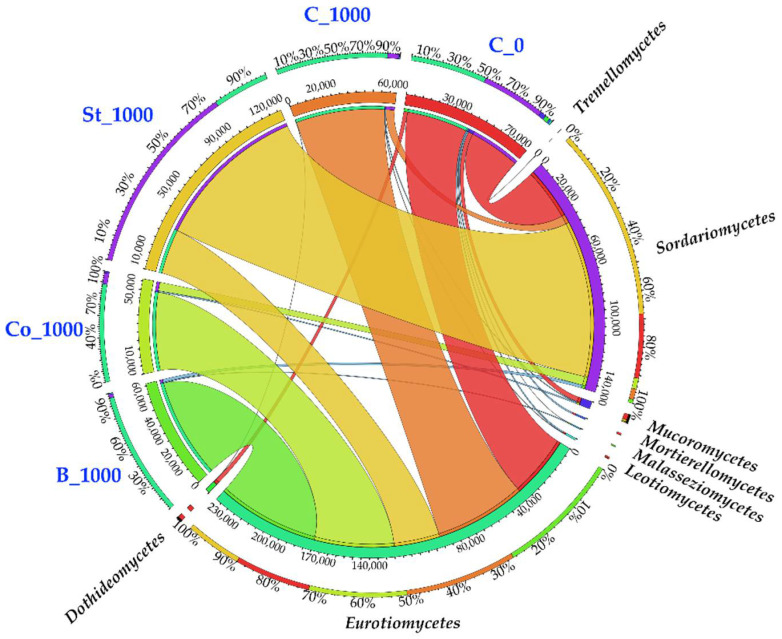
Dominant classes of fungi in soils. C_0—uncontaminated soil, St—starch, Co—compost, B—fermented bark, 1000—dose of BPA kg^−1^ d.m. of soil.

Changes in fungal structure at the phylum level were also significantly associated with diversity and abundance at the class level (Figure 6). The control soil sample contained eight fungal classes, including the Ascomycota classes *Eurotiomycetes*, *Sordariomycetes*, *Leotiomycetes,* and *Dothideomycetes*, which are classified as Ascomycota. These were followed by *Mucoromycetes* (Mucoromycota), *Mortierellomyces* (Mortierellomycota) and *Malasseziomycetes* and *Tremellomycetes* (Basidiomycota). The Eurotiomycetes (38,975 OTUs) and Sordariomycetes (32,729 OTUs) classes were the most abundant fungi. Notably, the application of BPA to the soil resulted in a 46.57% increase in *Eurotiomycetes* OTUs, ultimately causing this class of fungi to account for 89.92% of the biodiversity at this taxonomic level. Conversely, *Sordariomycetes* abundance decreased by 83.32% under xenobiotic pressure, reducing their representation from 41.42% to 8.59%. Notably, the phenolic compound eliminated three fungal classes entirely: *Mortierellomycetes*, *Malasseziomycetes,* and *Tremellomycetes*. Fermented bark was the most effective stimulator of *Eurotiomycetes* proliferation, as reflected by an increase in OTUs from 57,124 in BPA-contaminated soil to 63,733 in soil enriched with the tested organic material. Conversely, starch significantly increased the OTU value of *Sordariomycetes* fungi, rising from 5460 in soil under xenobiotic pressure to 98,281, effectively counteracting the phenolic compound’s negative impact.

**Figure 7:**
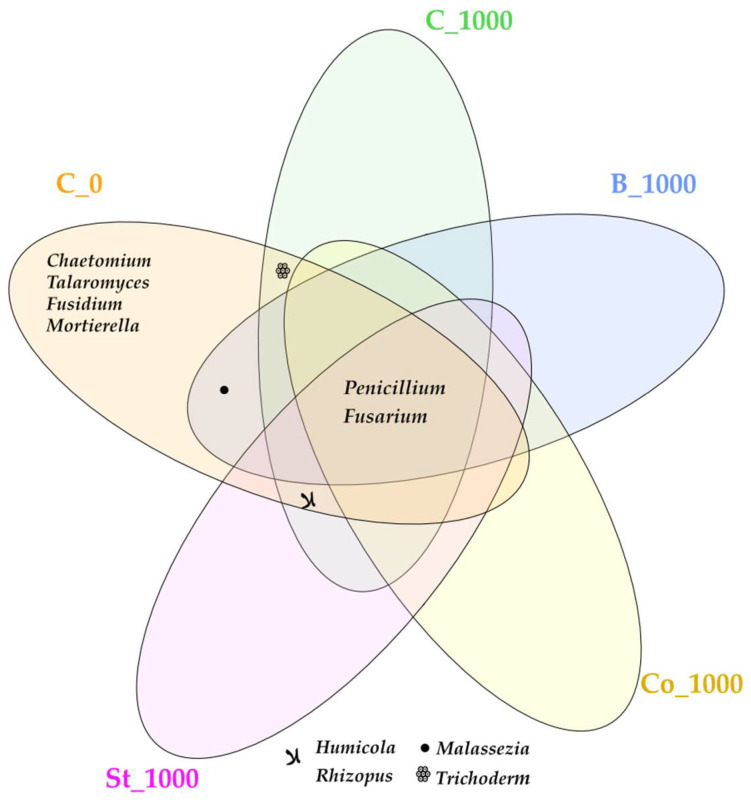
Unique and common fungal genera presented in the form of a Venn diagram. C_0—uncontaminated soil, St—starch, Co—compost, B—fermented bark, 1000—dose of BPA kg^−1^ d.m. of soil.

The sensitivity of the mold fungi assigned to identified classes resulted in the formation of a fungal profile at the genus level (Figure 7). Seven of the ten genera of fungi belong to the phylum Ascomycota: *Penicillium*, *Chaetomium*, *Talaromyces*, *Humicola*, *Fusidium*, *Fusarium*, *Trichoderma*, one genus *Mortierella* assigned to the phylum Mortierellomycota, *Rhizopus* to Mucoromycota, and *Malassezia* is a representative of the phylum Basidiomycota. Undoubtedly, the genus *Penicillium* proved to be the least sensitive to BPA soil contamination. The phenolic compound enhanced their proliferation, contributing to an 87.50% increase in OTU abundance compared to the control. BPA also positively affected the abundance of Fusarium OTUs, increasing them from 287 to 1904. For the other identified taxa, the escalation of BPA’s negative impact manifested as the elimination of their presence, with the exception of *Rhizopus*. Fermented bark increased in the proliferation of *Penicillium*, while starch reduced the inhibitory effect of BPA on *Humicola* by a spectacular amount. Starch stimulation generated an OTU abundance of 94,332.

**Figure 8:**
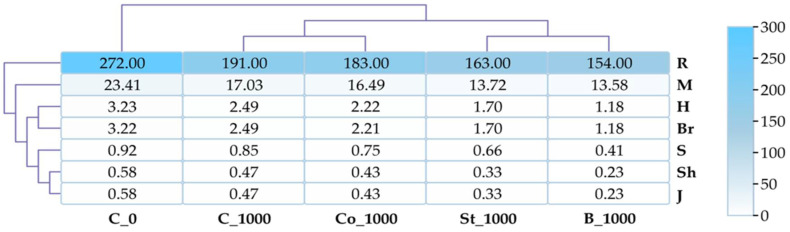
Fungal diversity indices Shannon (H), Simpson (S), Margalef (M), Brillouin (Br), and Richness (R); Pielou (J, Shavenen Sh); C_0—uncontaminated soil, St—starch, Co—compost, B—fermented bark, 1000—dose of BPA kg^−1^ d.m. of soil.

The calculated diversity indices for fungi indicated a stronger impact of BPA on this group of microorganisms than on bacteria (Figure 8). Application of BPA to the soil (C_1000) caused the following decreases compared to the control: Margalef index (27.25%), Shannon index (22.91%), Brillouin index (22.67%), Pielou index (18.96%), and Simpson index (7.61%). It should be emphasized that none of the applied organic materials induced an increase in the described parameter.

#### 2.3.3. Phospholipid and Ergosterol Composition of Soil

Soil lipids can serve as markers of microbial diversity and indicators of soil health. The percentage composition of polar lipids extracted from soil samples is presented in Table 1 and Table S2. Phosphatidylglycerol (PG) and phosphatidylcholine (PC) were the most abundant phospholipids in the control soil (C_0). In starch-amended soil, PC dominated (49.41%), while PE and PG occurred in nearly equal amounts (25–26%). A high content of phosphatidylglycerol (PG) content may indicate a high bacteria abundance. Such results were observed in control soil (C_0), bark-amended soil (B_1000), and grass compost-amended soil (Co_1000), with values of 41.34, 44.74, and 36.47, respectively. However, bacteria appeared to be more sensitive to BPA than fungi. The high proportion of phosphatidylcholine (PC) composed of 18-carbon polyunsaturated fatty acids in starch-amended soil also indicates a high abundance of fungi, which was 35% higher compared to the control. Ergosterol (E) is considered a reliable indicator of fungal presence. The results showed that the application of bisphenol A at a dose of 1000 mg BPA kg^−1^ d.m. of soil did not negatively affect fungal abundance. One possible explanation for this is the decrease in PG content, in both the percentage composition of phospholipids and the total PG content, after BPA application to soil. Conversely, the addition of organic materials such as compost and starch mitigated BPA toxicity to bacteria. The unsaturation index (UI) and the PC/PE ratio are useful indicators reflecting the fluidity of the cell membrane. In soil treated with a combination of BPA and starch, the unsaturation index (UI) increased to 1.25 compared to 1.02 in the control soil, and the PC/PE ratio increased to 2.00 from 1.10 in the control soil.

#### 2.3.4. Interdependence Between Membrane Lipid Composition and Indicators of Fungal and Bacterial Diversity

**Figure 9:**
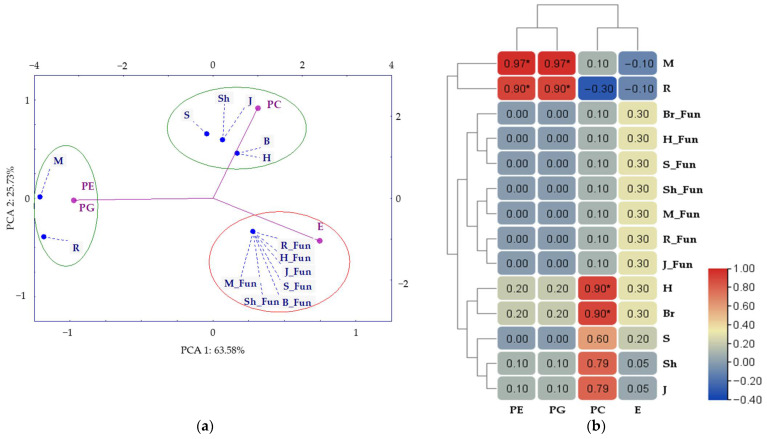
Principal component analysis (PCA) (**a**) and heat map showing Spearman’s rank correlations between phospholipids (PC, PE, and PG) and ergosterol (E) and the diversity indices of bacteria and fungi (Fun) (**b**). PE—phosphatidylethanolamine, PC—phosphatidylcholine, PG—phosphatidylglycerol, E—ergosterol, Shannon—H, Simpson—S, Margalef—M, Richness —R, Pielou—J—(Shaneven—Sh), Brillouin—Br. Statistically significant correlation coefficients (*p* < 0.05) are indicated by an asterisk (*).

Conducting a principal component analysis (PCA), conducted based on lipid parameters (PC, PE, PG, and E) and Spearman’s rank correlation coefficients relating bacterial and fungal diversity indices to these lipids, revealed significant relationships (Figure 9). The content of phosphatidylethanolamine (PE) and phosphatidylglycerol (PG) was significantly correlated with the bacterial Margalef (M) and Richness (R) indices (r = 0.97 and r = 0.90, respectively), suggesting that these lipids play a key role in determining the complexity and diversity of the bacterial microbiome.

## 3. Discussion

### 3.1. Zea mays

Analysis and discussion of the response of *Zea mays* is a necessary research step, particularly since phenolic compounds, including the five bisphenols identified as capilarisenols A–E, play a significant role in determining the chemical stability of plant cells [45]. This issue is also complex due to the multitude of processes involved in bisphenol transformation, such as glycosylation, glucosylation, hydroxylation, and redox reactions. These lead to the formation of secondary metabolites such as mono- and di-O-*β*-D-pyranosides [46]. Studies have also demonstrated that BPA is significantly more toxic to Zea mays roots than to its aboveground parts, which constitutes the main focus of the discussion (Figure S1). However, according to Zhao et al. [47] and Li et al. [48], doses of BPA below 5 mg kg^−1^ d.m. of soil promote corn root growth. This effect is mainly manifested by increased mitochondrial energy and cell proliferation in this part of the plant. It also results from maintaining reactive oxygen species (ROS) homeostasis associated with the induction of antioxidant enzyme activities: APX (ascorbate peroxidase), involved in neutralizing hydrogen peroxide (H_2_O_2_) in plant cells, and GPX (glutathione peroxidase), which reduces hydrogen peroxide (H_2_O_2_) using glutathione (GSH) as a substrate. However, many researchers [47,48,49,50,51,52] report adverse effects of BPA on *Zea mays* root growth. This phenomenon is signaled by genes, enzymes, and growth regulators whose activity is inhibited in the plant. Decreased expression has been observed in genes such as *EXPA8* and *EXPA10* [49], as well as in enzymes including glutamate dehydrogenase (GDH) [47], catalase (CAT), phosphofructokinase, and hexokinases [50]; and growth regulators including abscisic acid (ABA) [48], glutathione [53], and proline [47]. Ultimately, these changes lead to the induction of lipid peroxidation [54] and increased chromosomal aberrations. This results in damage to the microtubule matrix, causing it to undergo disruption. These processes affect the meristematic cells at the root tips [55]. In our studies, exposure to 500 and 1000 mg BPA kg^−1^ d.m. of soil caused reductions in root biomass of 57.74% and 86.11%, respectively. Therefore, *Zea mays* responded negatively when assessed by its aboveground parts, although the reduction in biomass was less pronounced at 44.11% and 69.75%, respectively (Figure S1). This can be explained by the lower fat content of stems and leaves compared to roots, and by the limited synthesis of conjugated estrogens resulting from the presence of a hydroxyl group in the BPA structure. This group is responsible for the mobility of this phenolic compound in corn [56]. The observed trends are further supported by the results of our previous studies [57], in which 1000 mg BPA kg^−1^ d.m. of soil contributed to a 64% reduction in corn root biomass.

The beneficial effect of compost on the growth and development of *Zea mays*, as well as its mitigation of the inhibitory impact of BPA on these parameters, can be attributed to the supply of essential macronutrients and micronutrients that support microbial activity [58,59]. Conversely, this compound contains complex organic substances such as sugars, lipids, proteins, and lignin. These are decomposed by bacteria belonging to the phyla Firmicutes, Proteobacteria, and Actinobacteriota into simpler forms that can be utilized by plants as a source of nutrients [60].

### 3.2. Culturable Bacteria

Tracking the response of soil microorganisms to BPA stress is a method of determining the extent to which the balance of the soil is disrupted. This disruption not only affects their activity, but also their biodegradation potential [40,61]. It is well-established that bisphenols reduce functional activity, and slow growth rates, as well as decreasing the intensity of microbial metabolic processes [62]. However, this does not apply to all groups. Studies have shown that organotrophic bacteria are the most sensitive to BPA soil contamination, while the abundance of fungi increased threefold under 500 mg BPA pressure (Table S1, Figure S2). Only their reproduction rate and ecophysiological diversity were inhibited and reduced, respectively. The F:B (Fun:Org/Act) ratio observed in our study, which is favorable for fungi, is an important ecological indicator of the soil microbiome’s response to pollution pressures, including BPA [63]. The importance of this ratio as a bioindicator of ecosystem response has also been emphasized by Sharma et al. [64]. Such strongly differentiated reactions in individual groups of microorganisms were observed thanks to high levels of contamination, at 500 and 1000 mg of BPA kg^−1^ d.m. of soil. This trend is corroborated by our previous research [65]. Interestingly, lower tolerance of actinomycetes and organotrophic bacteria was observed at 10 mg than at 100 mg BPA kg^−1^ d.m. of soil. This can be explained by the toxicity of BPA to bacteria resulting from strong interference with lipid synthesis [48]. This also involves impairment of the functions of the cell nucleus, endoplasmic reticulum, and mitochondria, ultimately leading to cell death [66,67]. The negative impact of BPA on the abundance of organotrophic bacteria and actinomycetes may result not only from the toxicity of the xenobiotic itself as well as from its metabolites. These include compounds such as p-hydroxyphenyl alcohol, 4-hydroxyacetophenone, 4-hydroxybenzoic acid, and 4-hydroxybenzaldehyde [68]. Due to the strong toxicity of hydroquinone, an intermediate BPA degradation product, a negative fungal response could also be expected [69]. However, this microbial group, similarly to actinomycetes, is rich in enzymes capable of transforming bisphenols and thus mitigating the adverse effects of their activity [42,43]. The potential of extracellular enzymes such as peroxidases and laccases, which generate highly reactive phenoxyl radicals is most commonly highlighted. Zhang et al. [70] demonstrated that laccase accounts for 78% of the efficiency of biodegrading this phenolic compound and is associated with enzyme conversion. Intracellular monooxygenase CYP hydroxylation also plays a significant role [71].

The focus of our research is to assess the effectiveness of organic materials in mitigating the toxic effects of BPA on soil microbial activity. Our investigations indicate that grass compost fulfilled its intended purpose. In soil under pressure from 1000 mg BPA kg^−1^ d.m. of soil, it enhanced the proliferation of organotrophic bacteria, actinomycetes, and fungi. When combined with 500 mg BPA kg^−1^ d.m. of soil, it also had a positive effect on fungal reproduction rates and ecophysiological diversity (Figure S2). Fermented bark was the second most effective organic material, mitigating the inhibitory effects of the lower BPA dose on the three microbial groups. Starch only improved only the condition of soil that was not contaminated with BPA, as indicated by the positive response of fungi and actinomycetes.

The observed relationships highlight the positive effects of compost, which stems from its nature as a biostimulating substance with high organic matter content. It also improves soil structure and water retention, thereby eliminating the risk of reduced soil biodiversity by forming soil aggregates [72]. These aggregates provide numerous interaction sites, primarily for the sorption of organic contaminants. Compost is also a source of microorganisms and, importantly, is biologically stable. By supplying nutrients, it induces the growth of the autochthonous microbiome activity [31]. According to Guo et al. [73] and De Corato et al. [74], organotrophic bacteria such as *Pseudomonas*, *Flavobacterium*, *Sphingobacterium*, *Bacillus*, and *Streptomyces* are representative of compost at the maturation stage. Research by Zaborowska et al. [57] shows that doses of 500 and 1000 mg BPA kg^−1^ dry weight of soil can inhibit the activity of dehydrogenases, enzymes closely dependent on the activity of living microorganisms, by 82% and 89%, respectively. The authors also highlighted the beneficial effect of grass compost on this parameter in soil contaminated with 500 mg of BPA and starch, and in facilities subjected to 1000 mg of BPA. According to Liu et al. [75], biochar is also an effective organic material, which degraded 31.93 mg of BPA kg^−1^ d.m. of soil by activating persulfate in the soil. Hussain et al. [76] also demonstrated the effectiveness of compost in removing BPA, with amounts decreasing by 99% within 45 days. This was due to the stimulation of the growth of Firmicutes, Pseudomonadota, and Bacteroidetes type microorganisms, which are capable of degrading this xenobiotic. In turn, in the studies by Loffredo et al. [77] observed that the application of compost to soils increases the distribution coefficient (Kd) of BPA by 176%, which generates lower toxicity of this xenobiotic for microorganisms. An advantage of bark as an adsorptive medium for organic contaminants is its outer layer, characterized by a rough, hygroscopic structure [33]. The accumulation of contaminants in bark involves complex mechanisms, including adsorption onto the porous, metabolically inactive outer bark as well as adsorption and complexation or chelation with organic compounds such as waxes in bark cracks [78]. These compounds also include lipophilic substances such as sterols, triterpenic acids, and fatty acids [79].

In turn, starch is distinguished by a unique structure with variable crystallinity and the presence hydroxyl groups, which predispose it to interactions with bisphenols. However, Supriyadi et al. [33] suggest modifying its configuration by, for example, by organized incorporation of aromatic units alongside hydroxyl groups, to obtain an effective adsorbent.

### 3.3. Non-Culturable Microorganisms

#### 3.3.1. Bacteria

As soil health is indicated by the functional diversity of its microbiome, the study also tracked changes in the structure of bacterial communities in this environmental medium under BPA pressure. It was found that this compound induced a spectacular increase in the abundance of ASVs of the phylum Proteobacteria by as much as 94.72% compared to the control. While Actinobacteriota were also dominant alongside Proteobacteria in all treatments, their abundance decreased by 48.45% after BPA exposure (Figure 1). Similar soil microbiome responses to BPA at the phylum level were also obtained by Siczek et al. [80]. An increase in the proportion of Proteobacteria may be a manifestation of a structural resistance mechanism. This is due to the fact that this type is mainly represented by Gram-negative bacteria, which are characterized by a higher isoelectric point (pH = 4–5) than Gram-positive bacteria. Consequently, the binding strength of binary xenobiotics, including phenolic compounds, is weaker than in Gram-positive bacteria [81].

In this study, genus-level rearrangements were found to be consistent with phylum-level changes. The bacterial genera with the highest ASV values were identified as follows: *Cellulosimicrobium* (19,842), *Caulobacter* (7110), *Rhodanobacter* (6793), *Sphingomonas* (3605), *Mucilaginibacter* (3848), and *Pseudomonas* (3270) represent the microbial response to BPA biodegradation in soil (Figure 3). Importantly, numerous studies [82,83,84] have confirmed the biodegradation potential of the bacterial genera also identified in our experiment.

Notably, *Cellulosimicrobium*, a representative of the class Actinobacteria, was identified as the dominant genus in treatments exposed to 1000 mg BPA kg^−1^ d.m. of soil. This is likely due to the fact that *Actinobacteria* activate a range of adaptive mechanisms in response to stress associated with the presence of a xenobiotic in soil. These include the production of siderophores and the activation of five systems regulating that regulate responses to reactive oxygen species (ROS), namely σB, σR–RsrA, OxyR, OhrR, and Rex [82]. Actinobacteria also synthesize exopolysaccharides (EPS) with negatively charged functional groups, including phenolic groups, which determine their role in effective bioremediation [83].

Researchers such as Thathola et al. [84], Shobnam et al. [85], and Tian et al. [86] have also isolated five species of *Pseudomonas* with the ability to degrade BPA: *Pseudomonas palleroniana* GBPI_508 [84], *Pseudomonas putida* G320 [85], *Pseudomonas* sp. PH7, *Pseudomonas aeruginosa* JI104, and *Pseudomonas stutzeri* [86]. They also reported stimulation of the proliferation of numerous *Sphingomonas* species (*Sphingomonas* sp. SO11, *Sphingomonas* sp. SO1a, and *Sphingomonas* sp. SO4a) under BPA pressure.

It should also be emphasized that *Achromobacter*, identified as a unique genus in our study, had previously been reported by Zhang et al. [87]. They also detected intermediate compounds of BPA degradation involving this bacterium, including p-hydroxybenzaldehyde, p-hydroxybenzoic acid, and p-hydroquinone, the latter being considered toxic to microorganisms. It should be emphasized that bacteria of the genus *Rhodanobacter*, including the *Rhodanobacter* AYS5 strain, also exhibit a significant ability to degrade xenobiotics, particularly 1,4-dioxane and tetrahydrofuran (THF), making them promising candidates for bioremediation applications [88].

Of all the organic materials used in BPA-contaminated soil, starch promoted the proliferation of the largest number of bacterial genera (Figure 3). However, given that starch has limited functional group diversity, low thermal stability, and is easily biodegradable, its adsorption capacity may be inadequate [89]. It probably acted as a selective factor favoring microorganisms for which starch serves as an easily accessible carbon source [90].

#### 3.3.2. Fungi

The conducted study also addressed the issue of fungal diversity. Their taxonomic structure at the phylum level was primarily determined by the presence of *Ascomycota* in all samples examined (Figure 4). Moreover, in response to the application of 1000 mg BPA kg^−1^ d.m. of soil, the abundance of *Ascomycota* increased by 5.56% compared to the control, resulting in a dominance of 98.66% of the total fungal community in this treatment. Such trends were anticipated, given that *Ascomycota* reportedly constitute approximately 65% of all fungi [91]. Their biodegradation potential is linked to intracellular metabolism of organic pollutants, defined as intracellular detoxification based on oxidation by cytochrome P450 CYP enzymes and conjugation reactions involving transferases [92]. The degradation of a wide range of contaminants is facilitated by the ability of *Ascomycota* to assimilate complex carbohydrates, which does not necessarily require prior hydrolysis [93]. The changes in fungal structure observed in the study, with a predominance of the Ascomycota phylum, can be interpreted as a mechanism for maintaining the functional resistance of the soil microbiome to BPA pressure. This is related to the use of BPA as a carbon source by saprotrophic fungi, especially those of the genus *Penicillium*, which have a highly developed antioxidant system, including the enzymes catalase and superoxide dismutase. These enzymes protect the cell from oxidative stress [94]. In addition, Shedbalkar et al. [95] showed that *Penicillium ochrochloron* can perform single-electron oxidation of bisphenols, which is possible thanks to the synthesis of lignin peroxidase.

Nevertheless, the enzymatic profile of representatives of this phylum is highly diverse, which complicates the identification of a single biodegradation pathway. Although in our research the dominance of *Ascomycota* in BPA-contaminated soil was reflected by an increased number of OTUs assigned to the *Eurotiomycetes* class (Figure 5), the indisputable representative whose proliferation was significantly stimulated by BPA was the *Penicillium* genus. The applied xenobiotic also positively influenced the abundance of OTUs classified as *Fusarium*, belonging to the *Sordariomycetes* class. *Penicillium chrysogenum* demonstrates the ability to degrade BPA via para-hydroxylation, in which 1,2,3-trihydroxybenzene is converted into maleic acid [93]. In the case of *Fusarium incarnatum*, the biodegradation potential is attributed to laccase and esterase activities, which catalyze the formation of intermediate phenol-alcohol compounds which are subsequently degraded through cleavage of C-C bonds to produce CO_2_ and H_2_O [96].

Fermented bark promoted the proliferation of fungi from the *Penicillium* genus, whereas starch stimulated the growth of fungi from the *Humicola* genus, which belongs to the *Sordariomycetes* class (Figure 6). These findings corroborate Leitão’s [93] observations indicating an 83% transformation of lignin, which results from the capacity of strains of this genus to produce enzymes such as mannanases, pectinases, and cellulases, enabling the utilization of organic compounds such as bark, as organic material. Besides lignin, bark contains suberin, cellulose, pectin, hemicelluloses, starch, and ash [33].

#### 3.3.3. Phospholipid and Ergosterol Composition of Soil

Significant changes in the composition of the main classes of phospholipids extracted from the soil were induced by both BPA itself and the applied amendments (Table 1 and Table S2). BPA at a dose of 1000 mg kg^−1^ d.m. of soil caused a significant increase in the percentage content of phosphatidylcholine (PC), which was correlated with a decrease in phosphatidylethanolamine (PE). It should be emphasized that PC is the principal membrane phospholipid found in eukaryotic organisms. Only about 15% of bacteria are capable of synthesizing this phospholipid [97]. In the BPA-exposed soil samples, a twofold decrease in phosphatidylglycerol (PG) content was also observed compared to the control, suggesting a reduction in the population size of bacteria for which PG is a key phospholipid present in the cell membranes of almost all bacteria. Literature data indicate that bisphenol A (BPA) affects the composition of microbial lipids, both their composition and the permeability of cellular membranes [98,99]. BPA also induced changes in the length and saturation of fatty acid chains in membranes, which in our study was reflected by changes in the unsaturation index (UI) [68,99]. It is believed that the hydrophobicity of a compound is directly correlated with its accumulation in phospholipid bilayers, and consequently with cell toxicity. The properties of BPA cause phospholipids to be pulled from bilayer membranes, eventually leading to pore formation [100]. Furthermore, alterations in the ratio of saturated to unsaturated fatty acids may reflect the impact of BPA on SCD-1 (stearoyl-CoA desaturase-1) activity. SCD-1 is an enzyme that converts saturated fatty acids into monounsaturated fatty acids (MUFAs), including oleic acid and palmitoleic acid [101]. Zeng et al. [102] demonstrated that BPA disrupts the metabolism of phosphatidylglycerols (PG) and fatty acids, in animal cells. Another bisphenol, 2,4-bisphenol S (2,4-BPS), was found to alter PG metabolism in the Gram-positive bacterium *Enterococcus faecalis* [103]. Contrary to our findings, most PG increased significantly under 2,4-BPS exposure, suggesting that this xenobiotic activates PG metabolic pathways. In our study, the decrease in PG content correlated with an increase in PC in soil exposed to bisphenol A (BPA) and appears to result from BPA’s toxic effects, which lead to a reduction in bacterial abundance in favor of fungal populations. However, Chen et al. [103] investigated pure bacterial cultures and observed an opposite trend in the bacterial lipidome.

Analysis of phospholipid profiles and ergosterol content confirmed that fungi are less sensitive to BPA than bacteria. This is evidenced by an increase in both PC and ergosterol levels in soil exposed to BPA. Ergosterol is the primary sterol found exclusively in fungal cell membranes and thus serves as a selective indicator of their presence. However, not all fungi exhibit high tolerance to BPA. Hąc-Wydro et al. [99] and Jasińska et al. [104] showed that BPA caused changes in fungal membrane lipid composition and induced oxidative stress, leading to lipid peroxidation.

Based on the analysis of results obtained in this study, it can be concluded that compost (Co) was an effective organic material for both bacteria and fungi, inducing a significant increase in the percentage content of PC (Table S2). PL analysis also highlighted the beneficial effect of starch (St) on fungal abundance. In soil amended with starch, a statistically significant increase in the unsaturation index (UI) and a marked increase in the PC/PE ratio were recorded, which may lead to increased membrane fluidity. This effect may be caused, on one hand, by the increased fungal abundance in this soil, resulting in elevated PC content and levels of polyunsaturated 18-carbon fatty acids, which also indicate favorable growth conditions for fungi [105]. On the other hand, it may reflect a microbial response to BPA presence; for example, bacteria exhibited changes in lipid profiles, including an increase in the proportion of unsaturated fatty acids as an adaptive response to bisphenol exposure [106].

**Figure 10:**
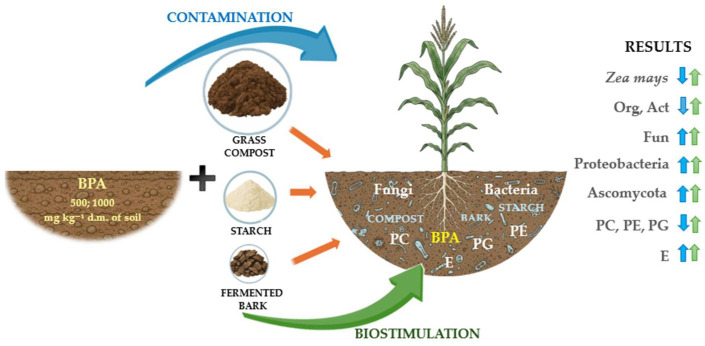
Graphical summary of the main results. ↑—increase in the value of the parameter studied, ↓—decrease in the value of the parameter studied, blue arrow—effect of BPA, green arrow—effect of organic materials. Cultivable microorganisms: Org—organotrophic bacteria, Act—actinomycetes, Fun—fungi: non-cultivable microorganisms: Proteobacteria, Ascomycota; PE—phosphatidylethanolamine, PC—phosphatidylcholine, PG—phosphatidylglycerol, E—ergosterol.

The results of the research are presented graphically in Figure 10.

## 4. Materials and Methods

### 4.1. Design and Procedure for Conducting Research on Soil Sown with Zea mays

The research objectives focused were to estimate changes in the structure of the soil microbiome to assess its sensitivity to the xenobiotic BPA. This generated a research procedure in which eliminating as many moderating factors as possible that could influence the expected results was the most important factor. The microbiome’s high sensitivity to soil imbalance, considered an important indicator [39] was prioritized. Consequently, the 60-day experiment was conducted in a 1035 m^2^ vegetation hall with advanced infrastructure, which guaranteed constant, maximum soil moisture level of 60%. Before packing the soil into pots (3.4 kg of soil each), it was sieved (mesh diameter 0.5 cm). The research factors established included the level of BPA contamination of the soil (0, 500 and 1000 mg BPA kg^−1^ d.m. of soil) and the selection of organic materials (control, starch, grass compost, and fermented bark). These constituted three series of four test objects, set up in four replicates: (1) objects with uncontaminated soil, (2) objects exposed to 500 mg BPA kg^−1^ d.m. of soil, and (3) objects exposed to 1000 mg BPA kg^−1^ d.m. of soil. All objects were enriched with remediating substances. Each was added to the soil at a rate of 20 g per kg of soil. All sites were also fertilized with macronutrients (N, P, K, and Mg) at doses corresponding to the optimal nutritional requirements of *Zea mays*, which were 150, 150, 50, and 20 mg kg^−1^ of soil, respectively. After mixing the phenolic compound, macronutrients, and organic materials with the soil, it was packed into pots and *Zea mays* L. was sown (8 seeds per pot). On the fifth day after the emergence of the coleoptile, the number of seedlings was reduced to four. On the 55th day, in accordance with the plant phase at which panicle emergence was observed (BBCH 51; Biologische Bundesanstalt, Bundessortenamt und Chemische Industrie (Federal Biological Research Centre for Agriculture and Forestry)), *Zea mays* was harvested to determine the yield of the aboveground and root parts. The choice of *Zea mays* was dictated by several significant facts. It was primarily determined by the complex root system of the plant, which facilitates the tracking of interactions in the rhizosphere [107]. It should also be taken into account that this plant not only plays a leading role in the feed industry, but also in the production of biofuels [108] and is an important food resource worldwide [109]. Thanks to a precise assessment of the soil microbiome’s response to high exposure, selected levels of BPA contamination allow the scale of point toxicity of soil contamination with this xenobiotic to be determined.

### 4.2. Characteristics of the Soil Sampling Area for Research

The soil used for analysis was collected from agricultural land in the Olsztyn Lake District that has been used for the long-term cultivation of cereals and fodder crops. These soils are ideal for agricultural use as they were formed mainly from clayey and sandy–clayey deposits. In the western part of the Masurian Lake District, agriculture is practiced on soils deposited on clayey moraine uplands. The diversity of land use is due to the fact that 40% of the area is covered by forests, primarily concentrated on sand sandurs. The lake district landscape also features numerous moraine hills, meltwater depressions and a dense network of lakes and waterways. A notable natural feature of the region is the Olsztyn Lake District Protected Landscape Area, which covers 40,997.4 hectares. This area is characterized by a moderately cool climate, with an average annual temperature of 6–7 °C and annual precipitation of 600–700 mm. However, during the year of the study, the average annual air temperature was 9.5 °C and total precipitation was 550 mm. The analyzed Eutric Cambisol soil, taken from a depth of 0–20 cm, is typical of intensively used agricultural habitats in young glacial regions and represents 1.5 million hectares worldwide [110].

### 4.3. Selected Chemical, Physicochemical, and Microbiological Properties of Soil

The first step of the research was to determine the granulometric composition of the soil. With a percentage content of 37.14% for sand, 54.71% for dust and 8.15% for clay, the soil was classified as silty clay with a pH of 6.7. Measurements were taken using the Malvern Mastersizer 3000 Laser Diffraction instrument (Malvern Panalytical Ltd., Malvern, UK) for granulometric composition and the HI 2221 pH meter (Hanna Instruments, Washington, UK) for soil pH [111]. The second step was to determine the soil’s selected chemical, physicochemical, and microbiological properties using standard testing methods (Table S1). Total nitrogen (N_Tot_) content was determined using the Büchi B-324 Kjeldahl Distillation Unit (Buchi, Flawil, Switzerland) [112]. The organic carbon content (C_org_) of the tested soil samples was determined with a TOC-5000 analyzer (Shimadzu, Kyoto, Japan) [113]. The Kappen method was used to determine hydrolytic acidity (HAC) and exchangeable base cations (EBC). The cation exchange capacity (CEC) and alkaline cation saturation (ACS) were determined using the Klute method [114]. As microbiological activity is an important soil parameter corresponding to its condition, the soil characteristics were expanded to include determining the abundance of organotrophic bacteria (Org) [115], actinomycetes (Act) [116] and fungi (Fun) [117].

### 4.4. BPA

Bisphenol A (BPA), with the chemical formula C_15_H_16_O_2_ and molecular weight 228.29 g mol^−1^, is a substance with a purity of ≥98.0% (HPLC), purchased from Sigma-Aldrich (CAS: 80-05-7). The parameters proposed in Table 2: soil adsorption coefficient (logK_OC_) vapor pressure (VP), water solubility (SW) and bioconcentration factor (BCF) are presented because they are most relevant to the mobility, adsorption, and migration potential of BPA in soil [19,118,119].

### 4.5. Characteristics of Organic Remediation Substances

The experiment identified the potential of three organic materials: starch (St) (Sigma Aldrich, Poznań, Poland), grass compost (Co) and fermented bark (B) (Athena Bio-Produkty Sp. z o.o., Golczewo, Poland). Their characteristics are as follows:

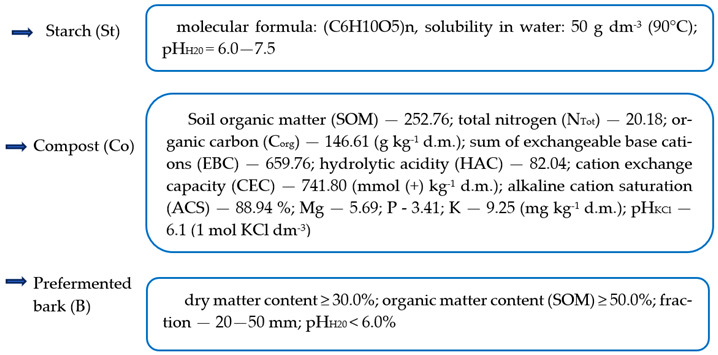



### 4.6. Methodology for the Determination of Cultivable Bacteria and Fungi

The selection of organotrophic bacteria (Org), which were grown on Bunta and Rovira medium [115], and actinomycetes (Act), which were grown on Küster and Williams medium [116], was dictated by the fact that these microorganisms play a key role in the circulation of organic matter in the soil among prokaryotic organisms [120]. Microscopic fungi (Fun), which are representatives of eukaryotic microorganisms, were also selected due to their role in forming aggregates and improving soil porosity and aeration [121]. This group of microorganisms proliferated on Martin’s medium [117]. The abundance of the three groups of microorganisms in each soil sample was determined by serial dilution (Org and Act: 10^−4^ and 10^−5^; Fun: 10^−2^ and 10^−3^) and deep plating, and is expressed in colony-forming units (CFU). Microbiological analyses were performed in four replicates. The colonies counted after 10 days of incubation at 28 °C (PSelecta Incubator, Barcelona, Spain) formed the basis for determining two key indicators: CD (colony development) and EP (ecophysiological diversity). These indices were calculated using the formulas presented by Sarathchandra et al. [122] and De Leji et al. [123].

### 4.7. Isolation of Bacterial and Fungal DNA

The MagnifiQ™ 1 Genomic DNA Instant Kit was used to isolate genomic DNA from soil samples. Mechanical lysis using zirconium balls with a FastPrep-24 was performed to effectively release DNA from microbial cells. The next stage of the analysis involved purifying the genetic material to remove any inhibitors that could potentially interfere with PCR and real-time PCR reactions. This was achieved using the Anti-Inhibitor Kit (A&A Biotechnology, Gdańsk, Poland). To accurately determine the amount of genetic material isolated, a fluorometric method (Qubit 4 Fluorometer) was employed. To confirm the presence of bacterial DNA, SYBR Green dye, which emits a fluorescent signal during amplification, was used. In this PCR reaction, universal primers were used to amplify a fragment of the bacterial *16S rRNA* gene. To confirm the presence of fungal DNA, *ITS1* and *ITS4* primers were used to amplify fragments of the *ITS* (internal transcribed spacer) region, which is characteristic of fungi.

### 4.8. Methodology for the Identification of Non-Culturable Bacteria and Fungi

To identify the bacteria present, the V3-V4 region of the 16S rRNA gene was amplified using the primers 341F and 785R. For fungi, the ITS1 (internal transcribed spacer) region was amplified using primers ITS1FI2 and 5.8S. The Illumina adapter sequences enabled the use of next-generation sequencing (NGS) technology. The amplification parameters for bacteria and fungi were the same, as was the sequencing process. The amplification reaction was performed using a thermocycler. The conditions for this were as follows: denaturation (98 °C for 30 s), annealing (25–35 cycles of 98 °C for 5–10 s and 50–72 °C for 10–30 s), extension (72 °C for 20–30 s) and a final extension step at 72 °C for two minutes. Sequencing on the Illumina MiSeq v2.6 platform yielded approximately 50,000 pairs of reads per sample (Genomed S.A. Warsaw, Poland). Following sequencing, the DNA reads underwent quality filtering and taxonomic assignment to species level using the QIIME 2 package and the GreenGenes v13_08 (bacteria) and UNITE v7 (fungi) reference databases. The microbial sequences were submitted to the National Center for Biotechnology Information under the following GenBank accession numbers: Prokaryotic *16S rRNA*: https://www.ncbi.nlm.nih.gov/nuccore/?term=OR032920:OR033134[accn] (accessed on 18 June 2023) and Eukaryotic Nuclear rRNA/ITS: https://www.ncbi.nlm.nih.gov/nuccore/?term=OR127278:OR128356[accn] (accessed on 28 May 2023). Seven indicators of bacterial and fungal diversity were determined based on metagenomic analyses: Shannon, Simpson, Margalef, Richness, Pielou (Shaneven) and Brillouin.

### 4.9. Analysis of Fatty Acid Profiles

#### 4.9.1. Soil Preparation

Soil samples (5 g) were extracted with 3 mL of methanol using a ball mill (Retsch, LMM 400, Haan, Germany) and glass beads (1 mm of diameter) for 8 min at 30 Hz of frequency. The samples were then centrifuged for 5 min at 2500× *g*. After filtered through filter paper, the supernatant was centrifuged once again in Eppendorf tubes (10 min, 9000× *g*).

#### 4.9.2. Lipids Analysis

A QTRAP 3200 mass spectrometer (Sciex Framingham, MA, USA) coupled with a 1200 series HPLC system (Agilent Technologies, Santa Clara, CA, USA) was used to measure the amount of ergosterol. A Kinetex C18 column (50 mm × 2.1 mm, particle size: 5 µm; Phenomenex, Torrance, CA, USA) was used for the separation. The solvents were methanol and water, each of which contained 5 mM ammonium formate. The QTRAP device had an atmospheric pressure chemical ionization (APCI) source that ran at 550 °C in positive ion mode. The monitored MRM pairs were *m*/*z* 379.3—69.1 and 379.3—81.3. Furthermore, cholesterol was used as the internal standard (*m*/*z* 369.3—147.2). The previous publications [124,125] provided detailed analysis conditions. A QTRAP 4500 mass spectrometer (Sciex, Framingham, MA, USA) equipped with an ESI source coupled with an ExionLC AC UHPLC system (Sciex, Framingham, MA, USA) was applied to phospholipids analysis. The mobile phase and column were the same as for the ergosterol analysis. The quantification of the amount of lipids in each class was completed through a comparison with the internal benchmark of the appropriate class. Details of the analysis were described earlier in the publications by Bernat et al. [124] and Rusetskaya et al. [125]. The specific class of phospholipids was shown as μg g^−1^ of soils and as a percentage of the overall amount of phospholipids. Based on the obtained data, the unsaturation index (UI) was calculated based on the following formula [124]:

$$UI=\frac{\left(\%C16;1+\%C18;1\right)+\left(\%C18;2\times 2\right)+(\%C18;3\times 3)}{100}$$

### 4.10. Calculation Methodology and Statistical Data Analysis

The toxicity of BPA to organotrophic bacteria (Org), fungi (Fun) and actinomycetes (Act), as well as its impact on the growth and development of *Zea mays*, was analyzed based on the BPA impact index (IF_BPA_). The effectiveness of the organic materials starch, grass compost and fermented bark on these parameters was also analyzed based on the established organic material impact index (IF_S_) values. PR ratio values were estimated based on the biomass of the above-ground parts of plants (P) and their roots (R). The IF_BPA_ and IF_S_ indices were calculated using the formulas described in the studies by Zaborowska et al. [52]. Principal component analysis (PCA) was used to determine the relationship between the studied variables and identify the main factors differentiating the analyzed samples. Tukey’s HSD test was used to compare means and identify homogeneous groups at a significance level of *p* = 0.05, taking into account the different sample sizes (N) [126]. STAMP 2.1.3 software [127,128] was used to analyze the relative abundance of dominant types of bacteria and fungi. The dominant classes were visualized using Circos 0.68 software [129]; unique and common types of microorganisms were presented using InteractiVenn software [130] (http://www.interactivenn.net/, accessed on 24 August 2025); and the Shannon–Weaver (H), Simpson (D), Margalef (Dm), Richness (R), and Brillouin (Br) indices. Correlations between phospholipids and ergosterol and these indices were visualized using the TBtools-II software v2.202 [131]. Based on the Shannon index (H’), the Pielou evenness index (J) and (Shaneven Sh) were calculated, both of which refer to the same phenomenon—the even distribution of individuals. Both names are given for clarity and reference in the sources. The values of the indices were calculated using the following formula [132]:

$$J\left(Sh\right)=\;\frac{H\prime }{lnS}$$

where

H′—Shannon Wiener index,
S—number of genera (genus richness).

The J-value (Sh) ranges from 0 to 1. A value of 1 indicates that individuals are distributed evenly among genera, while values closer to 0 indicate that one or more genera dominates the community.

## 5. Conclusions

The conducted research contributes to a better understanding of the extent of disturbances caused by BPA in soil. It highlights the importance of maintaining the balance and diversity of the microbiome as an element consistent with the principles of sustainable development. Although BPA promotes fungal proliferation, it was demonstrated that it exerts an inhibitory effect on the growth of organotrophic bacteria and actinomycetes. BPA also acts as a modulator of the diversity and structure of bacterial and fungal communities, as well as altering the content of phospholipids and ergosterol in soil, thereby disrupting the homeostasis of the soil environment. The observed trends are consistent with the adopted research hypothesis, confirming its validity.

In soil contaminated with 1000 mg BPA kg^−1^ d.m. of soil, this phenolic compound caused a decrease in the content of phosphatidylethanolamine (PE) and phosphatidylglycerol (PG), a key component of the cell membranes of nearly all bacteria. The bacterial community structure was primarily determined by microorganisms assigned to the phyla Proteobacteria and Actinobacteriota, which are more sensitive. A significant research achievement was the identification of bacteria from the genera *Cellulosimicrobium*, *Caulobacter*, *Rhodanobacter*, *Sphingomonas*, *Mucilaginibacter*, and *Pseudomonas* as responsive agents reflecting the potential for biodegradation of BPA in soil.

Analysis of selected soil lipid profiles and ergosterol further indicated that fungi are less sensitive to BPA than bacteria. Fungi were predominantly represented by the Ascomycota phylum, with the *Penicillium* genus being the least affected by this xenobiotic.

The application of organic materials such as starch, grass compost, and to a lesser extent, fermented bark, facilitated the restoration of soil microbiome balance, and confirmed our research hypothesis. Considering the holistic perspective of their effects, which underscores the significance of our findings, compost was identified as the most effective organic material among the three. It not only enhanced the proliferation of organotrophic bacteria, actinomycetes, and fungi but also positively influenced the growth and development of *Zea mays*, partially mitigating the inhibitory impact of BPA on this parameter.

## Figures and Tables

**Table 1:** Phospholipids composition (%, µg g^−1^ of soil) and ergosterol content (µg g^−1^ of soil) of soil methanolic extracts.

Lipids Content	C_0	BPA_1000	St_1000	Co_1000	B_1000
PC (%)	30.34 ^c^ ± 3.37	43.64 ^ab^ ± 1.85	49.41 ^a^ ± 3.87	40.54 ^b^ ± 2.63	19.34 ^d^ ± 1.61
PC (µg g^−1^ of soil)	0.34 ^b^ ± 0.03	0.33 ^b^ ± 0.02	0.64 ^a^ ± 0.01	0.36 ^b^ ± 0.01	0.15 ^c^ ± 0.01
PE (%)	28.04 ^b^ ± 0.68	25.25 ^bc^ ± 2.84	24.77 ^bc^ ± 0.75	22.93 ^c^ ± 0.72	35.86 ^a^ ± 3.47
PE (µg g^−1^ of soil)	0.26 ^a^ ± 0.05	0.17 ^c^ ± 0.04	0.22 ^b^ ± 0.02	0.20 ^b^ ± 0.02	0.25 ^a^ ± 0.00
PG (%)	41.34 ^ab^ ± 2.25	31.10 ^c^ ± 1.32	25.80 ^d^ ± 2.55	36.47 ^b^ ± 1.14	44.74 ^a^ ± 2.54
PG (µg g^−1^ of soil)	0.46 ^a^ ± 0.01	0.24 ^d^ ± 0.05	0.32 ^b^ ± 0.03	0.29 ^c^ ± 0.01	0.34 ^b^ ± 0.01
E (µg g^−1^ of soil)	1.33 ^b^ ± 0.12	1.69 ^a^ ± 0.18	1.88 ^a^ ± 0.07	1.30 ^b^ ± 0.17	0.80 ^c^ ± 0.09
UI	1.02 ^bc^ ± 0.05	1.10 ^b^ ± 0.03	1.25 ^a^ ± 0.08	1.06 ^bc^ ± 0.04	0.97 ^c^ ± 0.01
PC/PE	1.10 ^b^ ± 0.14	1.73 ^a^ ± 0.13	2.00 ^a^ ± 0.15	1.77 ^a^ ± 0.07	0.55 ^c^ ± 0.10

C_0—uncontaminated soil, St—starch, Co—compost, B—fermented bark, BPA—bisphenol A; 1000—dose of BPA kg^−1^ d.m. of soil, PE—phosphatidylethanolamine, PC—phosphatidylcholine, PG—phosphatidylglycerol, E—ergosterol Homogeneous groups marked with letters (a–d) were calculated separately for each phospholipid, ergosterol, UI—the unsaturation and PC/PE indexes.

**Table 2:** Selected four physicochemical properties of BPA determining its interactions in soil.

**Parameters**	**Terms/Values**	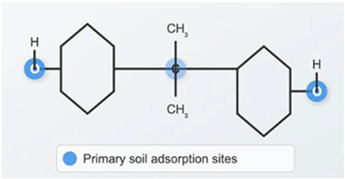
BPA synonyms	4,4′-isopropylidenediphenol 2,2-bis(4-hydroxyphenyl)-propane)	
log*K*_OC_	4.88	
*V*_P_ (Pa)	5.6 × 10^−6^	
*S*_W_ mg dm^−3^	120	
BCF	71.85	

## Data Availability

The original contributions presented in this study are included in the article/Supplementary Materials. Further inquiries can be directed to the corresponding authors.

## Supplementary Materials

The following supporting information can be downloaded at: https://www.mdpi.com/article/10.3390/molecules30193868/s1, Figure S1: The yield of Zea mays, aerial parts (P), roots (R) (f. m. g pot−1), (a); index PR (b)—ratio of aboveground parts to root biomass and IFBPA (c)—BPA influence index and IFS (d)—organic material influence index; Table S1: Microorganisms number per 1 kg d.m. of soil contaminated with BPA; Figure S2: The IFBPA (a)—BPA influence index and IFS (b)—organic material influence index on the abundance of organotrophic bacteria (Org), fungi (Fun), and actinomycetes (Act); Table S2: Phospholipids composition of soil methanolic extracts (%); Table S3: Some properties of the soil used in the experiment.

## Abbreviations

The following abbreviations are used in this manuscript: C—uncontaminated soil, BPA—bisphenol A, St—starch, Co—grass compost, B—fermented bark, PE—phosphatidylethanolamine, PG—phosphatidyl–glycerol, E—ergosterol, Org—organotrophic bacteria, Act—actinomycetes, Fun—fungi, CD—colony development index, EP—ecophysiological diversity index, H—Shannon diversity index, D—Simpson’s diversity index, Dm—Margalef’s diversity index, R—richness, J—Pielou’s evenness index (Shaneven Sh index), Br—Brillouin diversity index, IF_BPA_—BPA influence index, IF_S_—organic material influence index, P—aboveground part of plant, R—roots.
