# Supplementary material for: The Effect of Organic Materials on the Response of the Soil Microbiome to Bisphenol A
Source: Molecules. 2025 Sep 24;30(19):3868. doi: 10.3390/molecules30193868 (PMC12525994; doi:10.3390/molecules30193868)
Supplement: Supplementary file 1 [file molecules-30-03868-s001.zip › molecules-3864959-supplementary.pdf]

Supplementary Materials

The Effect of Organic Materials on the Response of the Soil Microbiome to Bisphenol A

Magdalena Zaborowska [1,*], Jadwiga Wyszkowska [1,*], Mirosława Słaba [2], Agata Borowik [1], Jan Kucharski [1] and Przemysław Bernat [2]

[1] Department of Soil Science and Microbiology, University of Warmia and Mazury in Olsztyn, Plac Łódzki 3, 10-727 Olsztyn, Poland;; agata.borowik@uwm.edu.pl (A.B.); jan.kucharski@uwm.edu.pl (J.K.)

[2] Department of Industrial Microbiology and Biotechnology, Faculty of Biology and Environmental Protection, University of Lodz, 90-237 Lodz, Poland; miroslawa.slaba@biol.uni.lodz.pl (M.S.); przemyslaw.ber-nat@biol.uni.lodz.pl (P.B.)

* Correspondence: jadwiga.wyszkowska@uwm.edu.pl (J.W.); m.zaborowska@uwm.edu.pl (M.Z.)

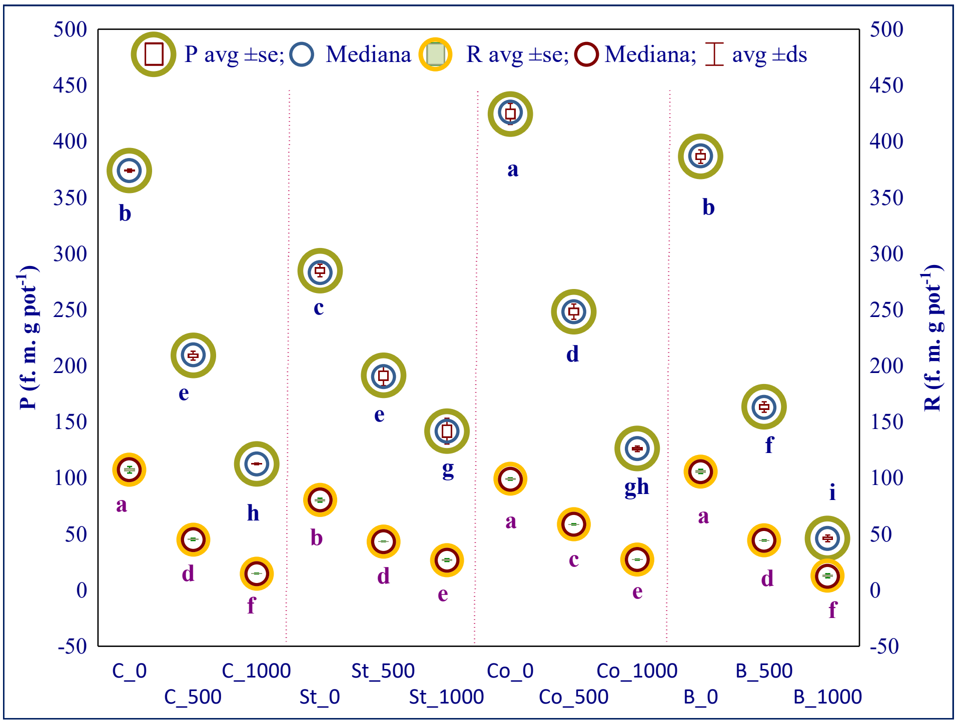

(a)

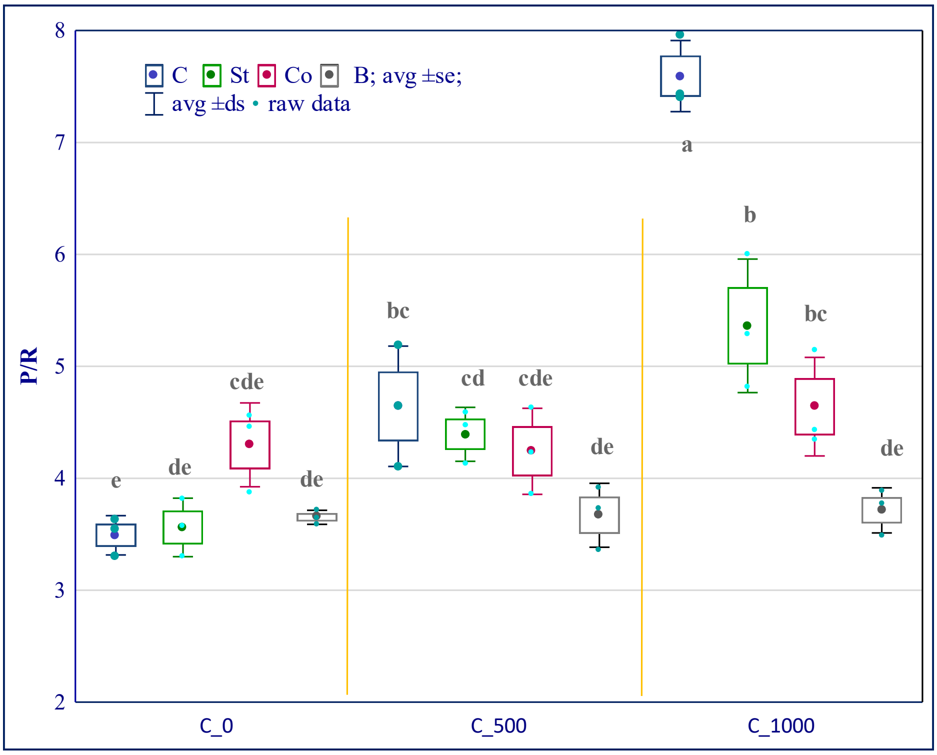
C St Co B; avg ±se;
avg ±ds • raw data
P/R
8
7
6
5
4
3
2
C_0
C_500
C_1000
a
b
bc
bc
cd
cde
cde
de
de
de
de
e

(b)

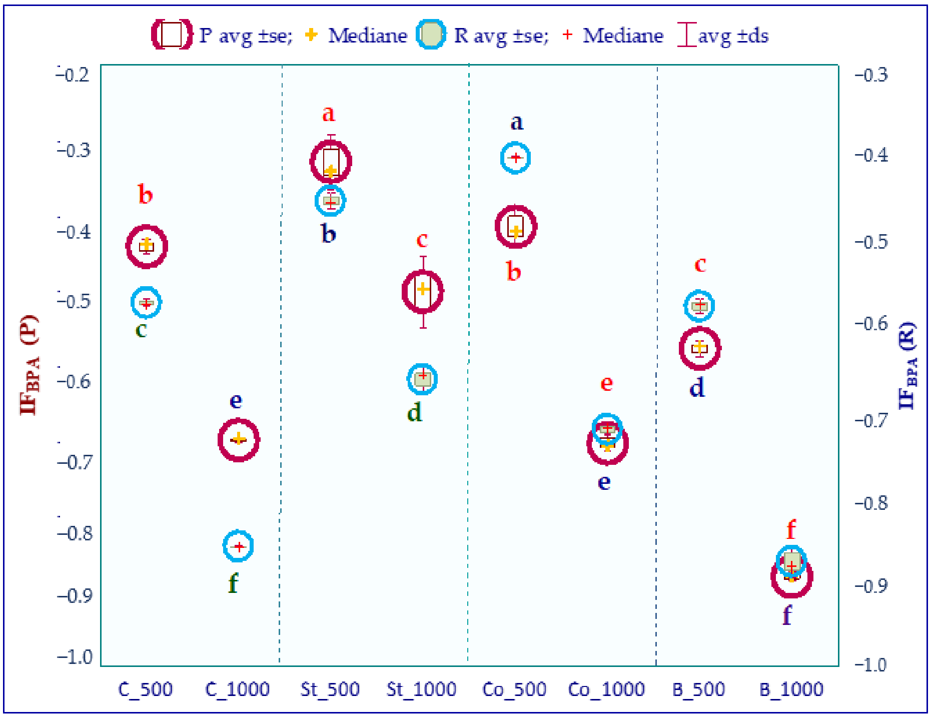
P avg ±se; Mediane R avg ±se; Mediane avg ±ds
IFBPA (P)
IFBPA (R)
C_500
C_1000
St_500
St_1000
Co_500
Co_1000
B_500
B_1000

(c)

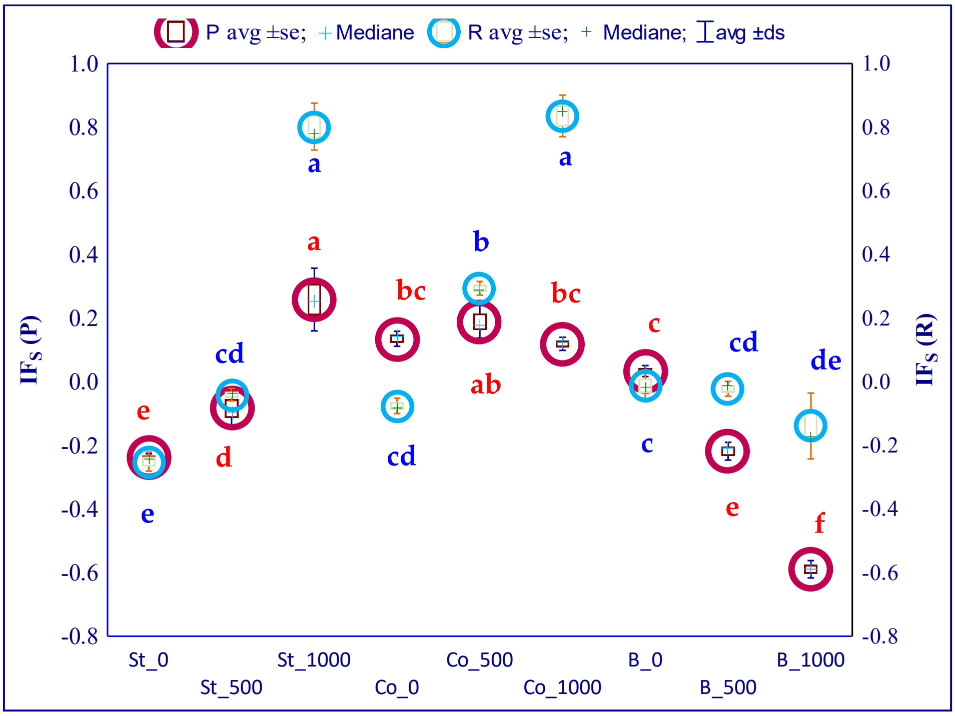

(d)

Figure S1. The yield of *Zea mays*, aerial parts (P), roots (R) (f. m. g pot^{-1}), (a); index PR– ratio of aboveground parts to root biomass and IF_{BPA} (b) — BPA influence index and IF_S (c) — organic material influence index(d); C_0 — uncontaminated soil, St — starch, Co — compost, B — fermented bark, BPA — bisphenol A; 500_BPA, 1000_BPA — doses of BPA kg^{-1} d.m. of soil. Homogeneous groups were determined individually for each parameter (represented respectively by the letters a—i (a); a—e (b), a—f (c, d)).

Table S1. Microorganisms number per 1 kg d.m. of soil contaminated with BPA

Object	Org 10^8	Fun 10^6	Act 10^8	Org	Fun	Act	Org	Fun	Act
	CFU kg^{-1}			CD			EP		
C_0	58.62±0.22^{a}	35.42±0.88^{c}	70.63±1.24^{a}	43.02±0.34^{a}	64.67±1.07^{a}	31.17±1.07^{a}	0.71±0.03^{b}	0.52±0.02^{a}	0.83±0.02^{b}
C_500	55.80±0.89^{b}	96.95±1.46^{a}	59.21±1.00^{c}	33.75±0.86^{c}	48.49±0.50^{b}	24.92±0.50^{b}	0.87±0.00^{a}	0.35±0.01^{b}	0.89±0.01^{a}
C_1000	38.50±1.15^{c}	65.19±0.54^{b}	64.06±1.09^{b}	37.67±0.43^{b}	47.48±0.38^{b}	25.96±0.38^{b}	0.87±0.01^{a}	0.35±0.02^{b}	0.89±0.02^{a}
average	50.97	65.85	64.63	38.15	53.55	27.35	0.82	0.41	0.87
St_0	134.20±1.65^{a}	124.86±1.34^{a}	140.11±0.63^{a}	59.17±0.54^{a}	54.80±0.38^{a}	39.29±0.38^{a}	0.67±0.02^{b}	0.44±0.01^{a}	0.75±0.01^{c}
St_500	81.65±0.93^{b}	125.17±1.90^{a}	60.87±0.49^{c}	38.60±0.47^{b}	52.51±0.10^{b}	27.54±0.02^{c}	0.86±0.02^{a}	0.44±0.01^{a}	0.81±0.01^{a}
St_1000	53.83±1.42^{c}	103.74±1.61^{b}	68.28±0.72^{b}	37.63±1.04^{b}	51.15±0.02^{c}	29.24±0.10^{b}	0.87±0.01^{a}	0.34±0.01^{b}	0.79±0.01^{ab}
average	89.89	117.75	89.75	45.13	52.82	32.02	0.80	0.41	0.78
Co_0	159.48±0.83^{a}	55.52±0.99^{c}	128.05±1.17^{a}	50.67±0.57^{a}	54.99±0.40^{b}	34.64±0.40^{a}	0.77±0.00^{c}	0.54±0.02^{a}	0.77±0.02^{b}
Co_500	102.40±0.88^{b}	97.40±1.39^{a}	118.45±1.24^{b}	42.68±0.60^{b}	58.08±0.57^{a}	25.37±0.57^{b}	0.89±0.00^{a}	0.44±0.01^{b}	0.88±0.01^{a}
Co_1000	94.35±0.51^{c}	89.30±1.82^{b}	83.54±1.11^{c}	43.63±0.37^{b}	51.47±0.46^{c}	24.31±0.46^{b}	0.86±0.00^{b}	0.42±0.01^{b}	0.86±0.01^{a}
average	118.75	80.74	110.02	45.66	54.84	28.11	0.84	0.47	0.84
B_0	95.56±0.99^{b}	39.87±0.97^{c}	76.52±1.23^{c}	46.62±0.87^{a}	55.03±0.97^{a}	35.66±0.97^{a}	0.88±0.01^{a}	0.46±0.02^{a}	0.74±0.02^{b}
B_500	149.43±1.09^{a}	117.07±1.66^{a}	140.48±0.81^{a}	36.55±0.30^{c}	45.98±0.37^{b}	26.92±0.37^{b}	0.86±0.01^{b}	0.28±0.02^{c}	0.86±0.02^{a}
B_1000	78.33±0.65^{c}	105.68±1.54^{b}	80.20±1.55^{b}	43.25±0.77^{b}	44.85±0.39^{b}	22.76±0.39^{c}	0.76±0.01^{c}	0.33±0.02^{b}	0.87±0.02^{a}
average	107.77	87.54	99.07	42.14	48.62	28.45	0.84	0.36	0.82

Org — organotrophic bacteria, Fun — fungi, Act — actinomycetes, C_0 — uncontaminated soil, St — starch, Co — compost, B — fermented bark, BPA — bisphenol A; 500_BPA, 1000_BPA — doses of BPA kg^{-1} d.m. of soil, CD — the colony development index, EP — the ecophysiological diversity index, homogeneous groups were determined individually for each parameter (represented respectively by the letters a — c (for CFU kg^{-1}; CD, and EP separately).

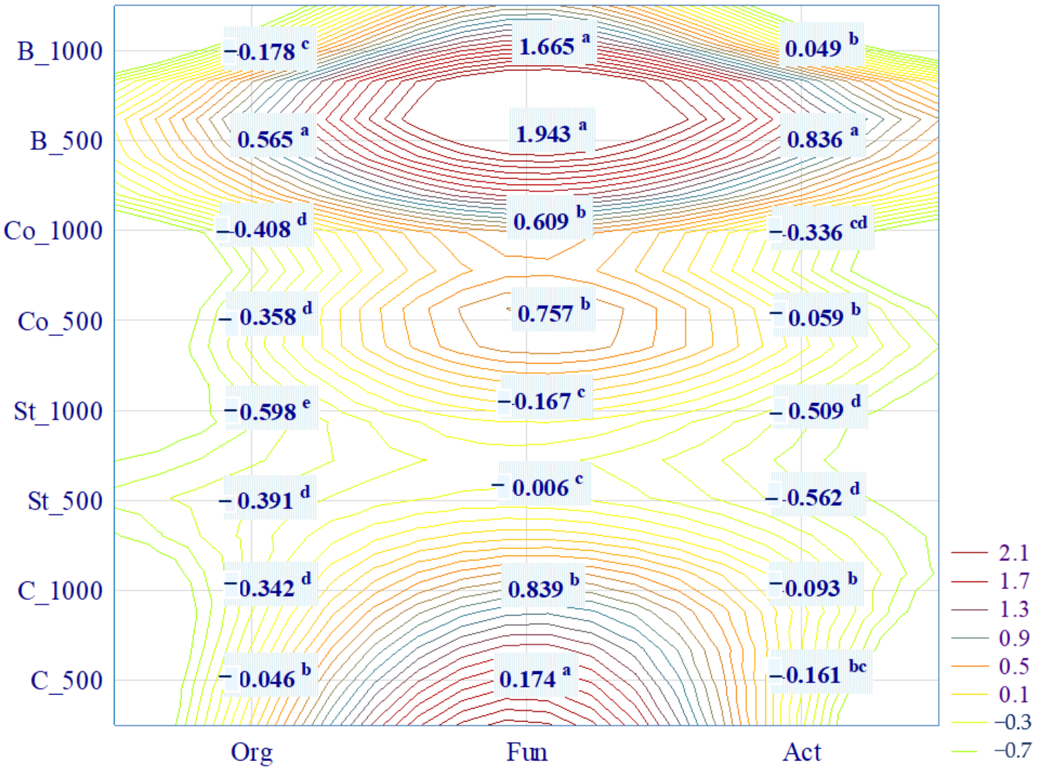

(a)

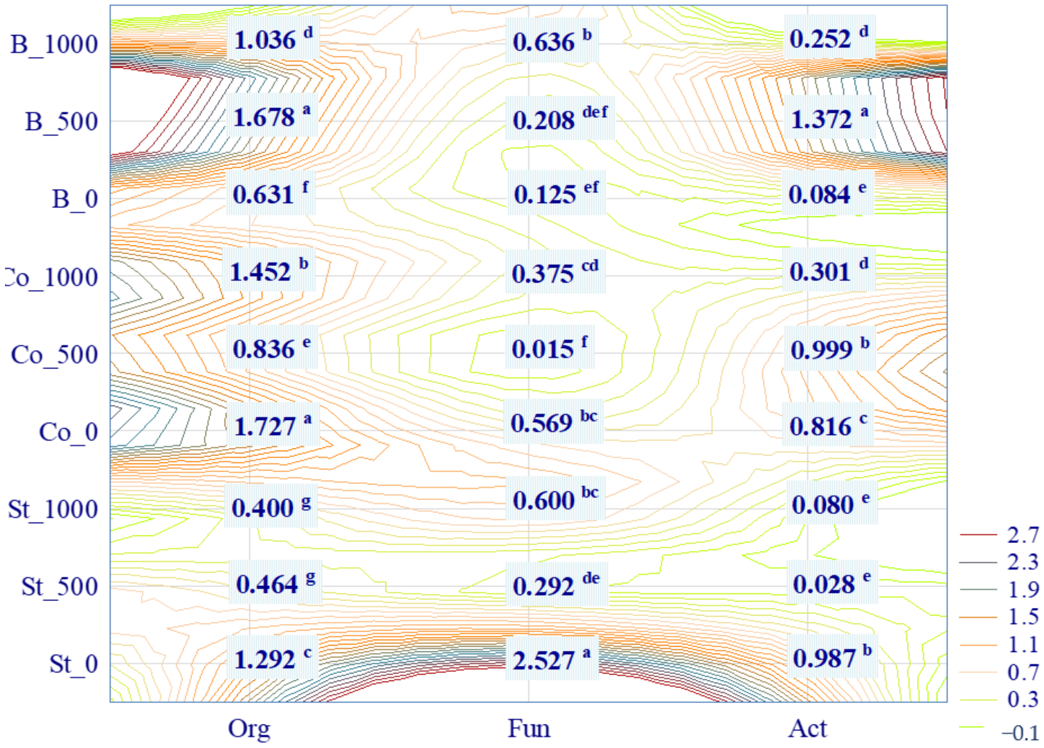

(b)

Figure S2. The IF_{BPA}— BPA influence index (a) and IF_S— organic material influence index on the abundance of organotrophic bacteria (Org), fungi (Fun), and actinomycetes (Act) (b); St — starch, Co — compost, B — fermented bark, BPA — bisphenol A; 500, 1000 — doses of BPA kg^{-1} d.m. of soil. Homogeneous groups were determined individually for each parameter (represented respectively by the letters a — e (a); a — g (b)).

Table S2. Phospholipids composition of soil methanolic extracts (%)

%	C_0	BPA_1000	St_1000	Co_1000	B_1000
PC 18;1 18;1	16.94 ^{b} ±1.89	23.67 ^{a} ±1.58	14.22 ^{c} ±1.34	21.94 ^{a} ±1.38	11.73 ^{d} ±0.66
PC 18;2 18;1	3.77 ^{c} ±0.77	6.66 ^{b} ±0.76	10.71 ^{a} ±1.01	6.84 ^{b} ±0.44	2.74 ^{c} ±0.41
PC 18;2 18;2	9.64 ^{c} ±1.23	13.31 ^{b} ±0.79	24.47 ^{a} ±1.82	11.75 ^{b} ±1.15	4.87 ^{d} ±0.54
PE 16;1 16;1	14.65 ^{b} ±1.52	11.42 ^{c} ±1.12	11.61 ^{c} ±0.89	12.35 ^{bc} ±0.92	21.29 ^{a} ±2.61
PE 18;1 18;1	4.27 ^{ab} ±0.28	5.06 ^{a} ±0.52	2.97 ^{b} ±0.64	3.86 ^{ab} ±0.36	3.69 ^{ab} ±0.72
PE 18;2 18;2	2.00 ^{bc} ±0.85	3.37 ^{a} ±0.45	2.92 ^{ab} ±0.36	1.38 ^{c} ±0.13	1.18 ^{c} ±0.22
PE 16;0 16;1	3.46 ^{a} ±0.33	3.39 ^{a} ±0.36	3.41 ^{a} ±0.27	3.46 ^{a} ±0.30	3.90 ^{a} ±0.75
PE 18;1 16;1	3.65 ^{a} ±0.54	2.01 ^{b} ±0.39	3.85 ^{a} ±0.81	1.89 ^{b} ±0.28	4.83 ^{a} ±0.76
PG 16;0 18;1	2.92 ^{b} ±1.08	4.87 ^{a} ±0.37	2.11 ^{bc} ±0.18	2.84 ^{b} ±0.29	1.52 ^{c} ±0.45
PG 18;1 18;1	8.71 ^{a} ±0.67	8.63 ^{a} ±0.53	4.67 ^{c} ±0.51	7.60 ^{ab} ±0.51	6.40 ^{b} ±0.63
PG 16:016:0	2.50 ^{b} ±0.43	2.18 ^{bc} ±0.43	1.88 ^{bc} ±0.38	4.35 ^{a} ±0.81	1.48 ^{c} ±0.18
PG 16:016:1	9.63 ^{b} ±1.18	6.61 ^{c} ±0.65	6.46 ^{c} ±0.58	7.06 ^{c} ±0.28	12.14 ^{a} ±1.37
PG 16:116:1	10.43 ^{b} ±1.33	5.17 ^{d} ±0.24	7.80 ^{c} ±0.58	9.11 ^{bc} ±0.80	13.88 ^{a} ±0.86
PG 18;1 16:1	7.15 ^{b} ±0.96	3.64 ^{d} ±0.19	2.89 ^{d} ±0.30	5.51 ^{c} ±0.95	9.32 ^{a} ±0.76

C_0 — uncontaminated soil, St — starch, Co — compost, B — fermented bark, BPA — bisphenol A; 1000 — dose of BPA kg^{-1} d.m. of soil. PE— phosphatidylethanolamine, PC — phosphatidylcholine, PG —phosphatidylglycerol. Homogeneous groups were determined individually for each phospholipid (represented respectively by the letters a—d).

Table S3. Some properties of the soil used in the experiment

Abbreviation	Unit	Value	Literature
Chemical and physicochemical properties			
N_{Tot}	$g \times kg^{-1}$ d.m.	1.16	[100]
C_{org}		6.28	[101]
C:N		5.42	
EBC	mmol (+) × kg^{-1} d.m.	196.00	[102]
HAC		17.25	[102]
CEC		213.23	[102]
ACS	%	91.91	[102]
pH	1 mol KCl dm^{-3}	6.70	[99]
Microorganisms number per 1 kg d.m. of soil			
Org	CFU	$58.62 \cdot 10^{8}$	[103]
Act		$71.90 \cdot 10^{8}$	[104]
Fun		$34.48 \cdot 10^{6}$	[105]

N_{Tot} — total nitrogen, C_{org} — organic carbon, EBC — sum of exchangeable base cations, HAC — hydrolytic acidity, CEC — cation exchange capacity, ACS — alkaline cation saturation, pH —pH_{KCl} — soil reaction, Org — organotrophic bacteria, Act — actinomycetes, Fun — fungi
